# Protein aggregates containing wild-type and mutant NOTCH3 are major drivers of arterial pathology in CADASIL

**DOI:** 10.1172/JCI175789

**Published:** 2024-02-22

**Authors:** Nicolas Dupré, Florian Gueniot, Valérie Domenga-Denier, Virginie Dubosclard, Christelle Nilles, David Hill-Eubanks, Christelle Morgenthaler-Roth, Mark T. Nelson, Céline Keime, Lydia Danglot, Anne Joutel

**Affiliations:** 1 Université Paris Cité, Institute of Psychiatry and Neuroscience of Paris (IPNP), INSERM U1266, Paris, France.; 2 Department of Pharmacology, College of Medicine, University of Vermont, Burlington, Vermont, USA.; 3 Institut de Génétique et de Biologie Moléculaire et Cellulaire, CNRS UMR 7104, INSERM U1258, Université de Strasbourg, Illkirch, France.; 4 Division of Cardiovascular Sciences, University of Manchester, Manchester, United Kingdom.; 5 GHU Paris Psychiatrie et Neurosciences, Hôpital Sainte Anne, Paris, France.

**Keywords:** Neuroscience, Vascular biology, Monogenic diseases, Mouse models, Neurological disorders

## Abstract

Loss of arterial smooth muscle cells (SMCs) and abnormal accumulation of the extracellular domain of the NOTCH3 receptor (Notch3^ECD^) are the 2 core features of CADASIL, a common cerebral small vessel disease caused by highly stereotyped dominant mutations in *NOTCH3*. Yet the relationship between NOTCH3 receptor activity, Notch3^ECD^ accumulation, and arterial SMC loss has remained elusive, hampering the development of disease-modifying therapies. Using dedicated histopathological and multiscale imaging modalities, we could detect and quantify previously undetectable CADASIL-driven arterial SMC loss in the CNS of mice expressing the archetypal Arg169Cys mutation. We found that arterial pathology was more severe and Notch3^ECD^ accumulation greater in transgenic mice overexpressing the mutation on a wild-type *Notch3* background (Tg*Notch3^R169C^*) than in knockin *Notch3^R170C/R170C^* mice expressing this mutation without a wild-type *Notch3* copy. Notably, expression of *Notch3*-regulated genes was essentially unchanged in Tg*Notch3^R169C^* arteries. We further showed that wild-type Notch3^ECD^ coaggregated with mutant Notch3^ECD^ and that elimination of 1 copy of wild-type *Notch3* in Tg*Notch3^R169C^* was sufficient to attenuate Notch3^ECD^ accumulation and arterial pathology. These findings suggest that Notch3^ECD^ accumulation, involving mutant and wild-type NOTCH3, is a major driver of arterial SMC loss in CADASIL, paving the way for NOTCH3-lowering therapeutic strategies.

## Abstract 2

Notch3ECD accumulation, involving WT and mutant Notch3ECD, drives arterial pathology in a cerebral small vessel disease, cerebral autosomal dominant arteriopathy with subcortical infarcts and leukoencephalopathy.

## Graphical abstract

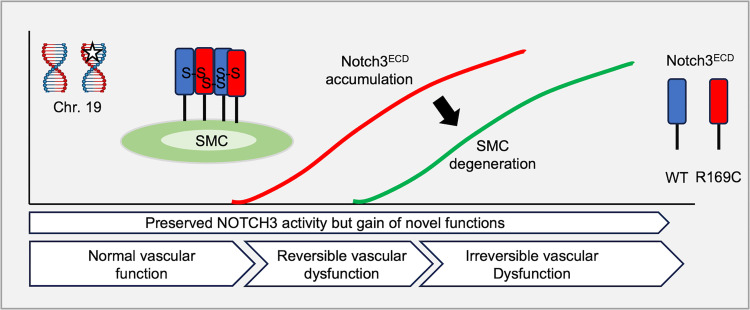

## Introduction

The *NOTCH3* gene encodes a receptor that is predominantly expressed in mural cells — pericytes and smooth muscle cells (SMCs) — of small blood vessels and is critically involved in the maturation, function, and survival of arterial SMCs (1). NOTCH3 signaling is initiated by the binding of transmembrane ligands to the NOTCH3 receptor, followed by sequential proteolytic cleavages of the receptor that release its intracellular domain into the nucleus, where it regulates transcription of SMC genes (2). Dominant mutations in human *NOTCH3* are the cause of CADASIL, the most common inherited form of cerebral small vessel diseases (cSVDs), characterized pathologically by the abnormal accumulation of granular osmiophilic material (GOM) around SMCs and pericytes of the brain microvasculature and the loss of SMCs in small brain arteries and arterioles (3–5). CADASIL-associated mutations invariably result in an odd number of cysteine residues in one of the 34 epidermal growth factor–like repeats (EGFrs) of the extracellular domain of NOTCH3 (Notch3^ECD^), leading to the abnormal aggregation and accumulation of Notch3^ECD^ in GOM deposits (6–10). The disease typically manifests as recurrent small strokes in deep brain regions at age 45–50 years, progressive cognitive decline, and, ultimately, premature death occurring 15–20 years later. To date, there are no available treatments that can prevent or slow the progression of the disease, imposing an enormous cost on society (11).

The development of disease-modifying therapies for CADASIL requires the unambiguous identification of the molecular disease mechanisms of pathogenic mutations. However, despite more than two decades of intensive research, the relationship between NOTCH3 receptor activity and Notch3^ECD^ accumulation on the one hand, and arterial SMC loss on the other, has remained elusive. The 2 most popular models to explain how NOTCH3 cysteine-altering mutations cause CADASIL-related arterial pathology, which are not mutually exclusive, are (a) a reduction in NOTCH3 signaling, herein referred to as a loss of function (LOF), and (b) a deleterious effect of Notch3^ECD^ aggregation that causes the mutant receptor to acquire a completely new function, herein referred to as a “neomorphic” effect (12). The first model is fueled by the identification (in 5%–10% of patients) of mutations in or around EGFr10–11 — the ligand-binding domain — that cause the mutant receptor to lose its signal transduction activity (13–16). For other mutations, which seemingly preserve normal activity, aggregation could similarly lead to LOF by destabilizing the mutant NOTCH3 and impairing its ability to transmit a signal. Moreover, if mutant NOTCH3 also induced wild-type (WT) NOTCH3 to aggregate, it could cause a more drastic reduction (>50%) in NOTCH3 activity, even though only half of the total protein synthesized is mutated. In keeping with this LOF hypothesis, genetic invalidation of *Notch3* in the mouse leads to arterial SMC loss in the cerebro-retinal vasculature and increased susceptibility to stroke (1, 17, 18). Moreover, biallelic null mutations in human *NOTCH3* are associated with a severe cSVD. Although distinct from CADASIL, this cSVD is characterized by early onset in infancy, the presence of livedo reticularis (a violaceous, netlike patterning of the skin) in half of patients, and an absence of GOM deposits (19–21). The second model, postulating a neomorphic effect, is supported by numerous studies on diseases associated with protein aggregation, where aggregates cause the disease by acquiring a novel, or even lethal, function (22). Consistent with this model is the finding that excess accumulation of the extracellular matrix metalloproteinase inhibitor TIMP3 in GOM deposits contributes to reversible cerebral blood flow deficits in a CADASIL mouse model by disrupting the activity of voltage-dependent potassium channels in SMCs (23–25). To date, however, definitive proof of either of these 2 models is lacking. Moreover, there are several other putative mechanisms by which CADASIL mutation could cause arterial pathology. Among features of these proposed alternatives are that aggregation of mutant NOTCH3 and pathogenesis occur independently or that Notch3^ECD^ aggregates mainly serve a protective function. It has also been hypothesized that pathogenesis may instead involve increased transcriptional activity of NOTCH3 (26).

The major impediment to the experimental studies needed to address this critical question is the failure to document arterial SMC loss in clinically relevant mouse models that express a cysteine-altering mutation (27) — a failure potentially attributable to the focal and segmental pattern of SMC loss in these models and technical difficulties in detecting it. We and others have shown that mice expressing a cysteine mutation in the EGFr10–11 ligand-binding domain backcrossed on a *Notch3*-null background do exhibit arterial SMC defects. However, while these experiments confirm that mutations in EGFr10–11 cause defective NOTCH3 function (16, 28), they provide no insight into the mechanism by which cysteine mutations cause arterial pathology, because such a genetic modification — homozygous null mutation in NOTCH3 — never occurs in CADASIL patients.

We designed the present study to disentangle which of these 2 mechanisms — an aberrant NOTCH3 signaling, i.e., a reduction or an increase in NOTCH3 activity, or a neomorphic effect — is involved in CADASIL-associated arterial SMC loss. We first tackled the problem of detecting brain arterial pathology in the central nervous system of clinically relevant CADASIL mouse models by using dedicated histopathological and imaging modalities. Further experiments using additional *Notch3*-related mouse models combined with histopathological, imaging, molecular, and genetic approaches demonstrated that, despite its strong resemblance to a loss of NOTCH3 function, CADASIL-associated arterial SMC loss is not driven by a loss of NOTCH3 activity; neither is it caused by an increase in NOTCH3 activity. Instead, our results provided compelling evidence that the abnormal accumulation of Notch3^ECD^, a process that involves both mutant and WT NOTCH3, is a major driver of arterial pathology. Our findings identify NOTCH3-lowering approaches as candidate therapeutic strategies and are thus expected to have a fundamental impact on the design of disease-modifying therapies.

## Results

### Conceptualization of the experimental strategy.

**Figure 1:**
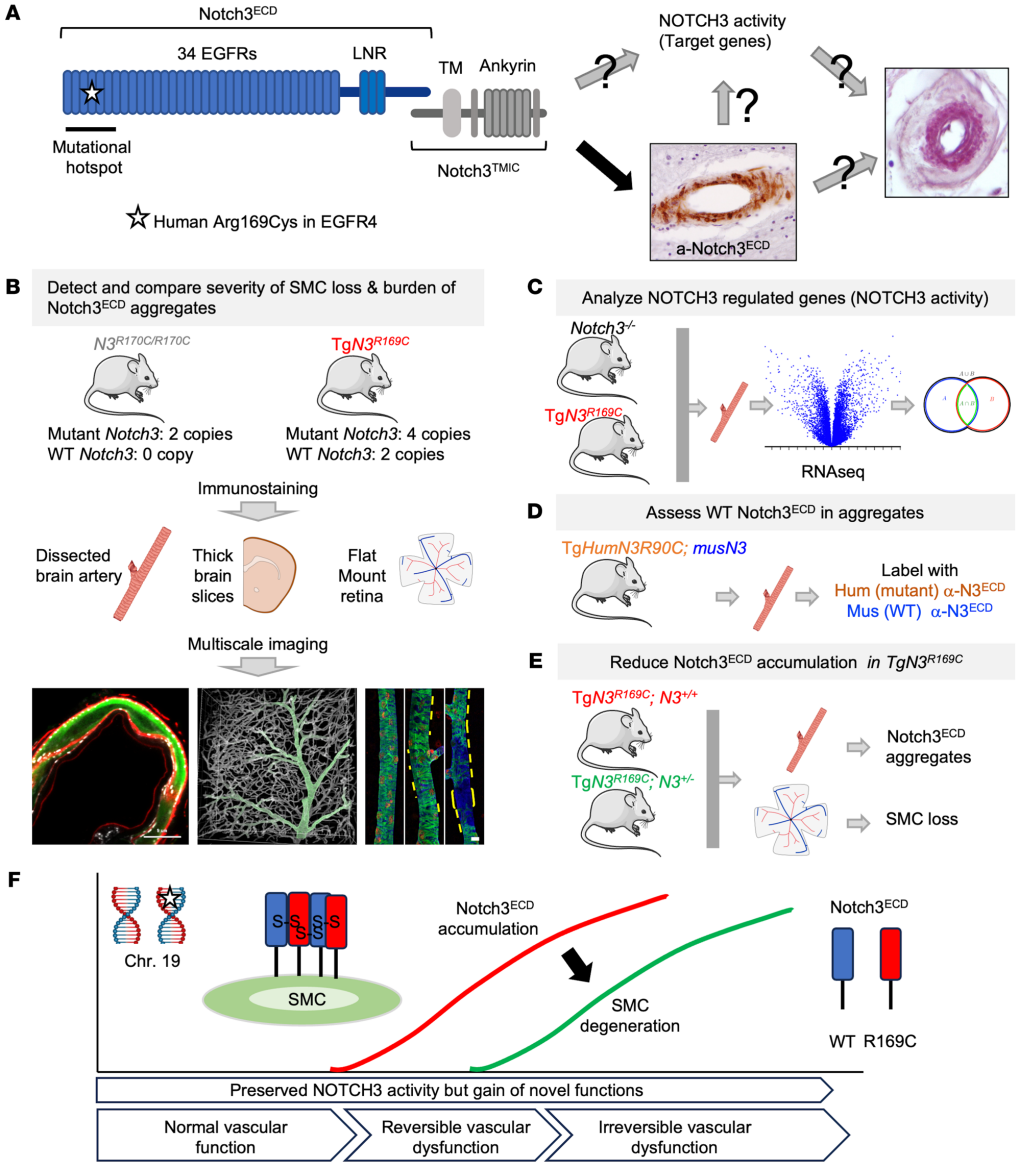
Schematic overview of the experimental strategy and working model. (**A**) Mechanisms put forward to explain how the Arg169Cys mutation causes arterial pathology. (**B**–**E**) Four main types of experiments used to discriminate between a loss/gain of NOTCH3 activity and a neomorphic effect. (**F**) Proposed model emerging from this study. NOTCH3 activity is unaffected and arterial maturation and vascular function proceed normally despite a CADASIL mutation, but mutant Notch3^ECD^ aggregates with WT Notch3^ECD^, possibly via a mechanism involving thiol disulfide exchange reactions. This leads initially to reversible vascular dysfunction owing to an excess of TIMP3, which compromises activity of K_v_2.1 voltage-dependent K^+^ channels (23–25). Upon reaching a threshold, accumulated Notch3^ECD^ aggregates cause arterial SMC loss and irreversible vascular dysfunction, possibly through accumulation and inactivation of other proteins (e.g., HTRA1).

As a representative mutation commonly found in CADASIL patients, we studied the human Arg169Cys mutation in EGFr4; this mutation, located in a mutational hotspot (EGFr1–6), is associated with a severe and highly penetrant phenotype (Figure 1A) (29). We used 2 distinct mouse models — the *Notch3^R170C^* and Tg*Notch3^R169C^* lines — that express a distinct number of copies of WT and mutant *Notch3* with this mutation (Figure 1B). The *Notch3^R170C^* line is a knockin model harboring a cysteine mutation inserted at codon 170 in the mouse *Notch3* gene that corresponds to the human Arg169Cys mutation (30). Mice were bred to homozygosity (*Notch3^R170C/R170C^*) to express 2 copies of the endogenous mutant *Notch3* gene at normal levels, and no copies of WT *Notch3*. This homozygous mutation is clinically relevant since several CADASIL patients with a homozygous cysteine mutation in EGFr1–6 have been described, particularly in Finland, where there is a founder effect (31). The phenotype of these patients is within the clinical spectrum of CADASIL, although at its more severe end (32). In contrast, the Tg*Notch3^R169C^* line is a transgenic model, created using a phage P1–derived artificial chromosome, that overexpresses rat *Notch3* containing the Arg169Cys mutation (4 copies) but also overexpresses normal murine *Notch3* (2 copies) (33). The *Notch3^R170C^* and Tg*Notch3^R169C^* lines, initially developed on a mixed SV/129-BL/6 background (*Notch3^R170C^*) or FVB/N (Tg*Notch3^R169^*) background, were backcrossed onto the same C57BL/6 background, based on recent work suggesting that the C57BL/6 background may be more permissive for the expressivity of cSVD phenotypes (34).

With these 2 mouse models in hand, we were poised to determine which of the 2 proposed mechanisms accounts for arterial SMC loss in CADASIL. Our approach was to compare the burden of Notch3^ECD^ accumulation and the severity of arterial pathology between Tg*Notch3^R169C^* and *Notch3^R170C/R170C^* mice and profile the transcriptome of NOTCH3-regulated genes (Figure 1, B and C). If arterial SMC loss is caused by a reduction in NOTCH3 activity, the prediction was that arterial pathology would be more severe in *Notch3^R170C/R170C^* mice, which lack WT NOTCH3. If instead arterial loss is caused by a neomorphic effect or an increase in NOTCH3 activity, arterial pathology should be more severe in Tg*Notch3^R169C^* mice, which would presumably also show greater aggregation and accumulation of Notch3^ECD^. In the latter scenario, gene expression profiling should enable discrimination between a neomorphic effect and increased NOTCH3 activity. To perform this phenotypic comparison, we developed dedicated histopathological and multiscale imaging modalities that allowed us to precisely quantify Notch3^ECD^ accumulation in dissected brain arteries and — more importantly — to identify and quantify arterial SMC defects in brain and retinal arteries. To further investigate the contribution of a neomorphic effect, we examined whether WT NOTCH3 participates in the accumulation process and assessed the impact of reducing Notch3^ECD^ accumulation on arterial pathology (Figure 1, D and E).

### Notch3^ECD^ aggregates and accumulates to a greater extent in TgNotch3^R169C^ than in Notch3^R170C/R170C^ mice.

**Figure 2:**
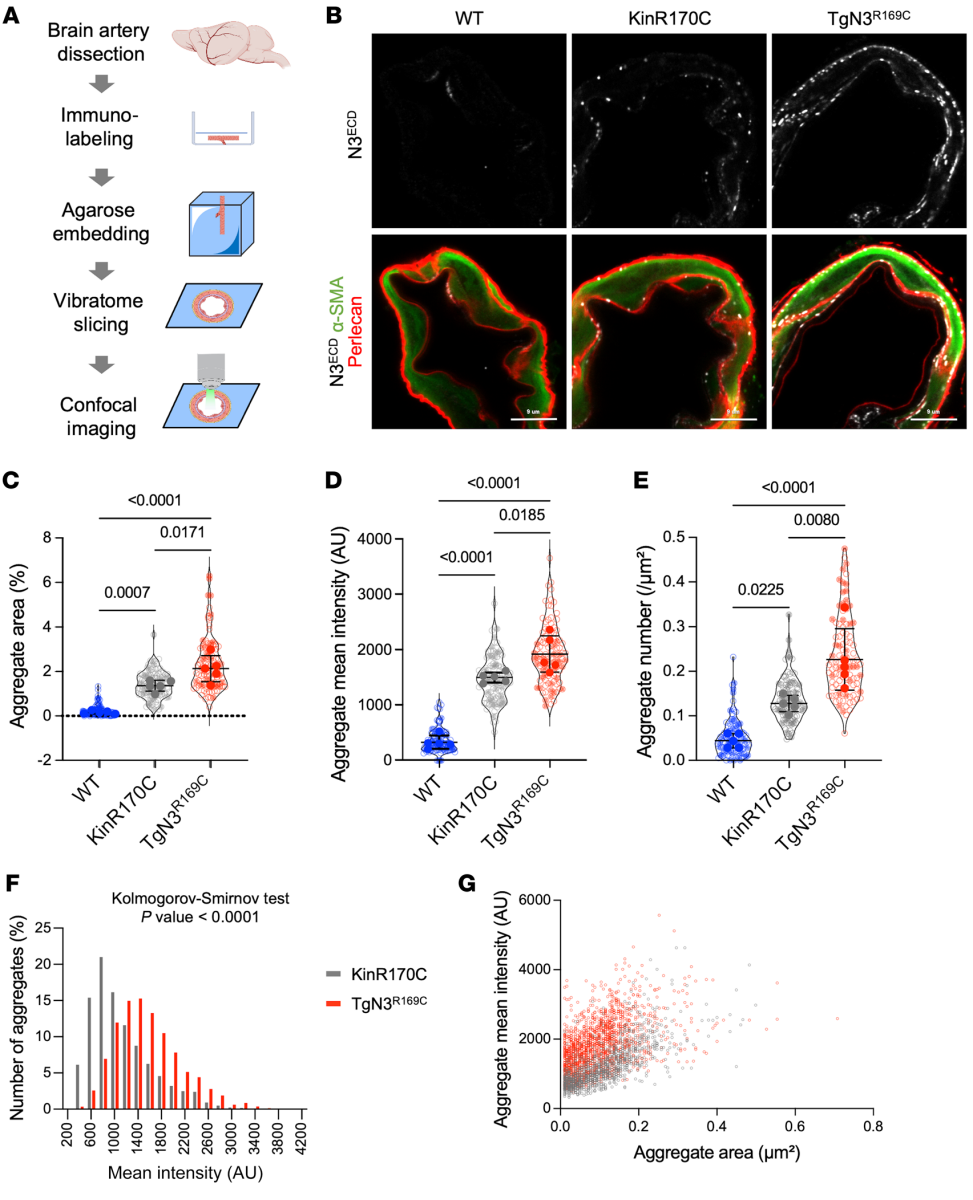
Notch3^ECD^ accumulation is greater in Tg*Notch3^R169C^* than in *Notch3^R170C/R170C^* mice. (**A**) Schematic overview of the protocol for labeling dissected brain arteries. (**B**) Representative confocal images of brain arteries from WT, *Notch3^R170C/R170C^* (KinR170C), and Tg*Notch3^R169C^* (TgN3^R169C^) mice aged 4 months, stained for α-SMA, perlecan, and Notch3^ECD^. (**C**–**E**) Quantification of the mean area (**C**), mean intensity (**D**), and number (**E**) of Notch3^ECD^ aggregates in brain arterial SMCs of WT, KinR170C, and TgN3^R169C^ mice (*n* = 5 mice per genotype with 20 SMCs per mouse). Shown are violin plots (individual data points represent individual SMCs) and scatter dot plots (individual data points represent individual animals). Data from individual animals were analyzed by 1-way ANOVA and Tukey’s post hoc test. (**F** and **G**) Intensity distribution of individual Notch3^ECD^ aggregates (**F**) and intensity versus size distribution of Notch3^ECD^ aggregates (**G**) in brain arteries of KinR170C and TgN3^R169C^ mice. Data were analyzed by Kolmogorov-Smirnov test. Scale bars: 9 μm (**B**).

We first assessed the burden of Notch3^ECD^ aggregation and accumulation between Tg*Notch3^R169C^* and *Notch3^R170C/R170C^* mice. Brain arteries from both models were dissected at 4 months of age, labeled in toto, and imaged using high-resolution confocal microscopy (Figure 2, A and B). Measurements of Notch3^ECD^ immunostaining in individual SMCs showed that both the area and the mean fluorescence intensity of immunostaining were significantly higher in Tg*Notch3^R169C^* mice compared with *Notch3^R170C/R170C^* mice (Supplemental Figure 1; supplemental material available online with this article; https://doi.org/10.1172/JCI175789DS1). Because these 2 metrics do not discriminate between a global increase in Notch3^ECD^ expression and genuine aggregation of Notch3^ECD^, we re-ran the image analysis protocol using more stringent parameters, adjusted so as to predominantly detect Notch3^ECD^ aggregates. This analysis confirmed the previous analysis (Figure 2, C and D), establishing that Notch3^ECD^ accumulation is more pronounced in Tg*Notch3^R169C^* mice. In WT mice, a few spots were detected along the internal elastic lamina, likely corresponding to clusters of NOTCH3 receptors expressed on myoendothelial projections (Figure 2B). Using this approach, we were also able to determine the number of Notch3^ECD^ aggregates as well as their size and mean intensity distribution per SMC. The 2 most pronounced differences between Tg*Notch3^R169C^* and *Notch3^R170C/R170C^* mice were the significantly higher number of aggregates and increased proportion of brighter aggregates (i.e., aggregates containing a higher number of Notch3^ECD^ molecules) in Tg*Notch3^R169C^* mice (Figure 2, E and F). Interestingly, this finding was not associated with a greater proportion of larger aggregates (Figure 2G). Collectively, these results confirm that the Notch3^ECD^ aggregates and accumulates to a greater extent in Tg*Notch3^R169C^* than in *Notch3^R170C/R170C^* mice.

### Detection of arterial defects in the brains of CADASIL mice is enabled by the use of thick brain slices.

Next, we tackled the problem of detecting brain arterial pathology in CADASIL mouse models. Pathological analyses of postmortem brain tissues from CADASIL patients have shown prominent loss of SMCs in small brain arteries, a hallmark of the disease (35, 36). Specifically, reconstruction of 1,000 serial paraffin sections from an autopsy subject revealed a diffuse loss of SMCs along the entire length of frontal cerebral medullary arteries from the penetrating site at the cortical surface to the distal end in the white matter (37). However, to date, we and others have failed to convincingly demonstrate the presence of arterial lesions in the brains of mouse models of CADASIL (30, 33, 38). Here, we suggest the possibility that arterial SMC loss is focal and segmental at the early stage of the disease and, assuming that this is recapitulated in mouse models, might go undetected by an analysis of a limited number of 5- to 10-μm-thick sections using conventional histological techniques. Thus, rather than using cross sections, we sought to image long arterial segments by immunostaining thick brain slices.

**Figure 3:**
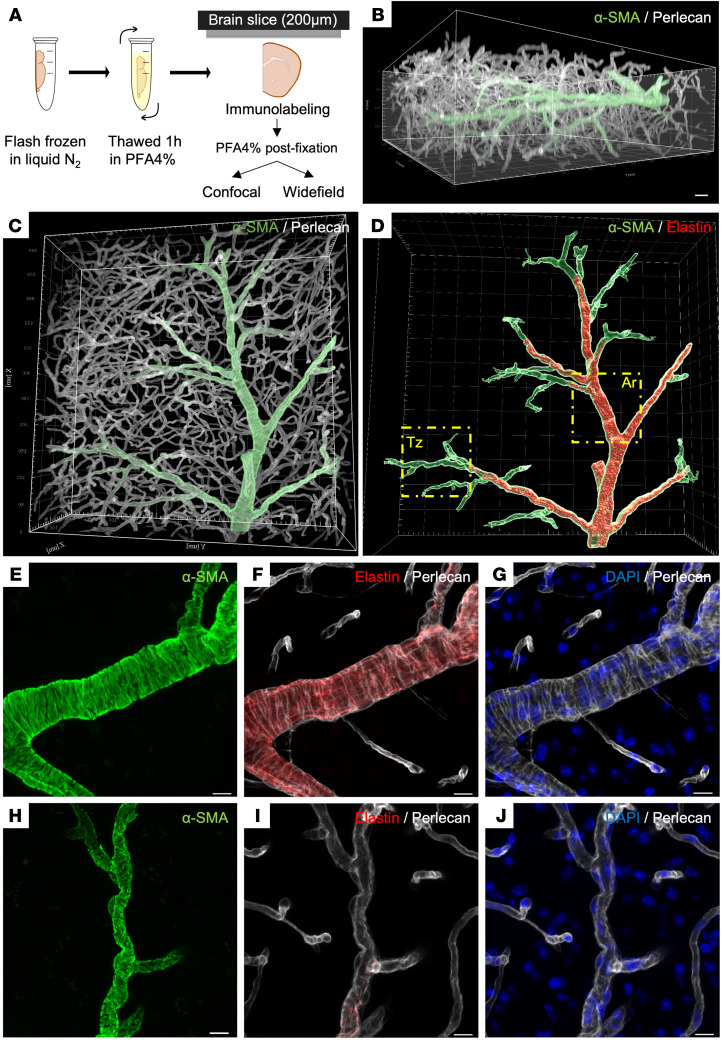
Thick brain slices enable the analysis of long arterial segments. (**A**) Schematic overview of the protocol starting with a frozen hemisphere thawed for 1 hour in 4% paraformaldehyde (PFA) and sliced at 200 μm thickness for immunostaining, then postfixed in PFA 4% for 15 minutes before imaging. (**B**–**J**) Confocal images of a 200-μm-thick brain section from a WT mouse stained for α-SMA (green), elastin (red), and perlecan (white), acquired at medium (194 × 194 × 750 nm) (**B**–**D**) or high (78 × 78 × 160 nm) (**E**–**J**) resolution. (**B** and **C**) 3D rendering views (Imaris software, Bitplane) showing staining of vessels throughout the entire thickness of a slice (**B**) and long α-SMA^+^ vascular segments within a single slice (**C**). (**D**) Segmentation of the arterial (elastin^+^ and α-SMA^+^) and transition zone (elastin^–^ and α-SMA^+^) compartments (Imaris software). (**E**–**J**) High-magnification images of an arteriole (Ar) (**E**–**G**) and transition zone (Tz) segment (**H**–**J**) delineated in panel **D** (yellow outlines), illustrating the distinct shapes of arterial SMCs and transition zone pericytes. Scale bars: 30 μm (**B**–**D**) and 10 μm (**E**–**J**).

Starting with coronal brain sections, we selected a thickness of 200 μm, a depth that allows imaging by different modalities without optical preclearing and over a wide range of pixel size resolutions (Figure 3A). Brain sections were labeled with antibodies against smooth muscle α-actin (α-SMA), a contractile protein expressed by SMCs; perlecan, a major component of vascular basement membranes; and elastin, a marker of the internal elastic lamina, which separates endothelial cells from SMCs in arteries/arterioles. To improve antibody penetration and immunoreactivity, we optimized every step, from fixation to immunostaining, as described in detail in Methods. Three-dimensional (3D) confocal imaging confirmed that antibodies did indeed penetrate throughout the entire thickness of the section (Figure 3B). Imaging at medium resolution (194 × 194 × 750 nm) showed elaborate vascular (perlecan-positive) networks that could be divided into 3 distinct microvascular segments according to their positivity or negativity for α-SMA and elastin labeling (Figure 3, C and D). Following the convention of recent studies (39–41), microvascular segments positive for α-SMA and elastin are considered arteries/arterioles; post-arteriole microvascular segments positive for α-SMA but negative for elastin are termed “transition zone” segments; and distal microvascular segments negative for both α-SMA and elastin are referred to simply as capillaries (Figure 3D). We then confirmed that the processing of brain samples and sections did not affect the integrity of mural cells. Imaging at higher resolution (78 × 78 × 160 nm) showed that SMCs of arteries/arterioles were ring-shaped, whereas mural cells in the post-arteriole regions had more irregular ensheathing processes with weaker α-SMA staining and protruding nuclei (Figure 3, E–J). Because acquisition of a single 581 × 581 × 185 μm field of view by confocal microscopy at medium resolution required approximately 1.5 hours and yielded an image file size of approximately 12.4 gigabytes, this imaging modality was deemed unsuitable for large-scale screening of SMC defects in multiple brain slices from dozens of animals. Seeking a compromise among acquisition, resolution, vessel-sampling, and data-handling criteria, we found that imaging with an epifluorescence microscope equipped with a motorized *xyz* stage and a ×5 (0.16 numerical aperture) objective provided a cellular resolution (1.3 μm pixel size) sufficient to assess the integrity of arterial SMCs in a coronal section of a whole hemisphere in only 3 minutes with a relatively low image size (200–300 megabytes) (Supplemental Figure 2). Using this approach, we found that the length of all arteries analyzed in 4 coronal sections of a single hemisphere totaled 127.5 ± 13.1 mm.

**Figure 4:**
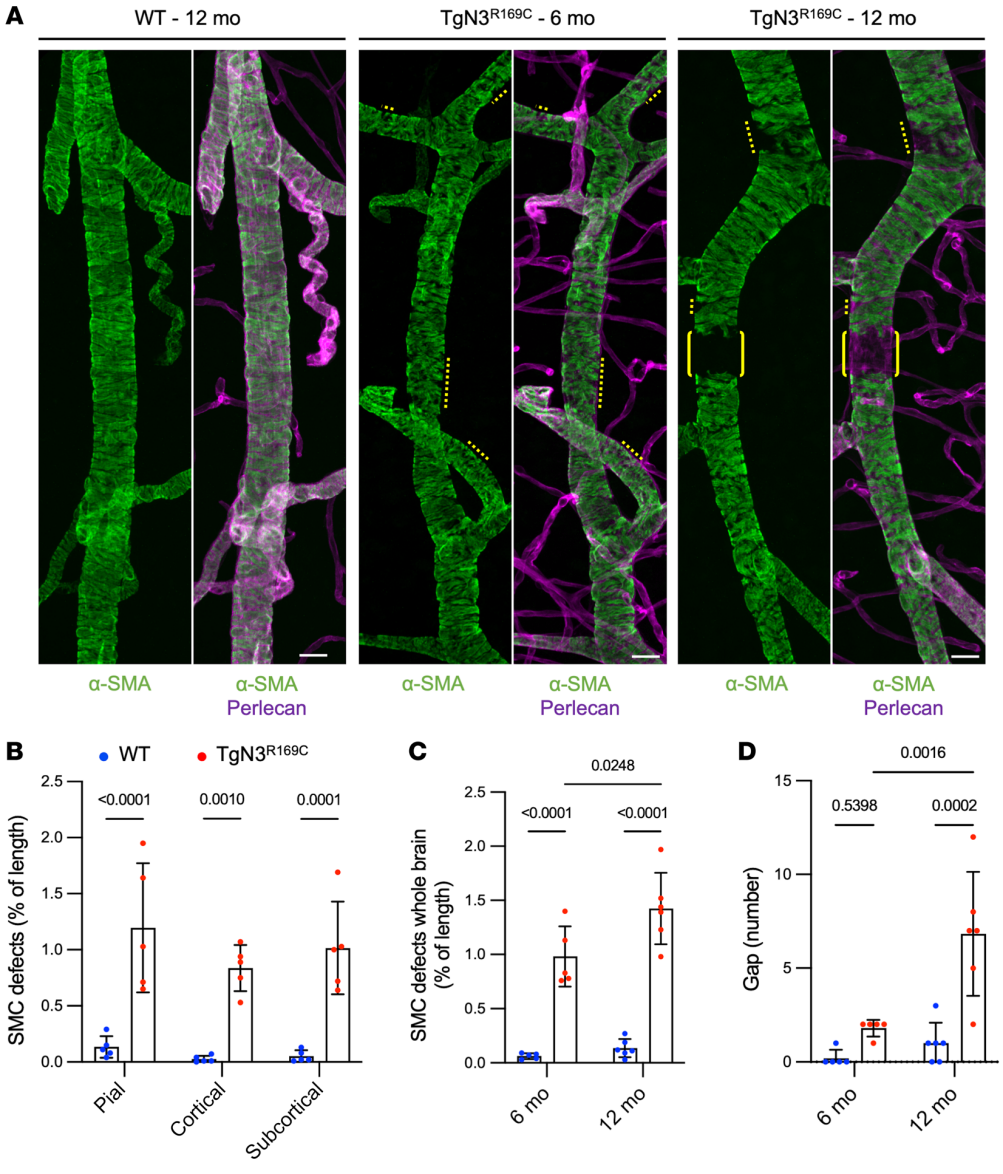
Brain arteries of Tg*Notch3^R169C^* exhibit arterial SMC defects. (**A**) Confocal images of 200-μm-thick brain sections stained for α-SMA, elastin, and perlecan. Shown are representative subcortical brain arteries from WT and Tg*Notch3^R169C^* (TgN3^R169C^) mice. Arteries from TgN3^R169C^ mice aged 6 months and older exhibit SMC defects, characterized by discontinuous or notched α-SMA staining (yellow dotted lines) as well as gaps in α-SMA staining (brackets). (**B**) Quantification of SMC defects in WT and TgN3^R169C^ mice aged 6 months in pial, cortical, and subcortical arteries (*n* = 5 mice per genotype). Data were analyzed by 2-way ANOVA and Šidák’s post hoc test. (**C** and **D**)Quantification of SMC defects (**C**) and SMC gap numbers (**D**) in brain arteries of WT and TgN3^R169C^ mice at 6 and 12 months (*n* = 5–6 mice per genotype). Data were analyzed by 2-way ANOVA and Tukey’s post hoc test. Scale bars: 20 μm (**A**).

We next assessed the integrity of brain arteries from Tg*Notch3^R169C^* and *Notch3^R170C/R170C^* mice, examining 4 coronal sections per animal, segmented into pial, cortical, and subcortical regions. We focused our analyses on arterial/arteriolar (α-SMA–positive and elastin-positive) segments. Arterial SMC integrity was comparable between *Notch3^R170C/R170C^* and age-matched WT mice up to 22 months of age (Supplemental Figure 3). In striking contrast, a comparison of Tg*Notch3^R169C^* mice and WT mice revealed discrete regions where α-SMA staining was looser or discontinuous, hereafter called “defects,” in 6-month-old Tg*Notch3^R169C^* mice that were not found in WT mice (Figure 4A and Supplemental Figure 4). Quantitative analyses of the lengths of these defects showed that pial, cortical, and subcortical arteries were similarly affected in mutant mice (Figure 4B) and that these defects worsened with age (Figure 4C). Specifically, 12-month-old Tg*Notch3^R169C^* mice harbored multiple gaps in α-SMA coverage — defined as arterial areas negative for α-SMA but positive for perlecan over the entire circumference — whereas fewer such gaps were detected in 6-month-old Tg*Notch3*^R169C^ mice (Figure 4D).

### Both TgNotch3^R169C^ and Notch3^R170C/R170C^ mice develop age-dependent arterial SMC defects in the retina, but defects are more severe in TgNotch3^R169C^ mice.

**Figure 5:**
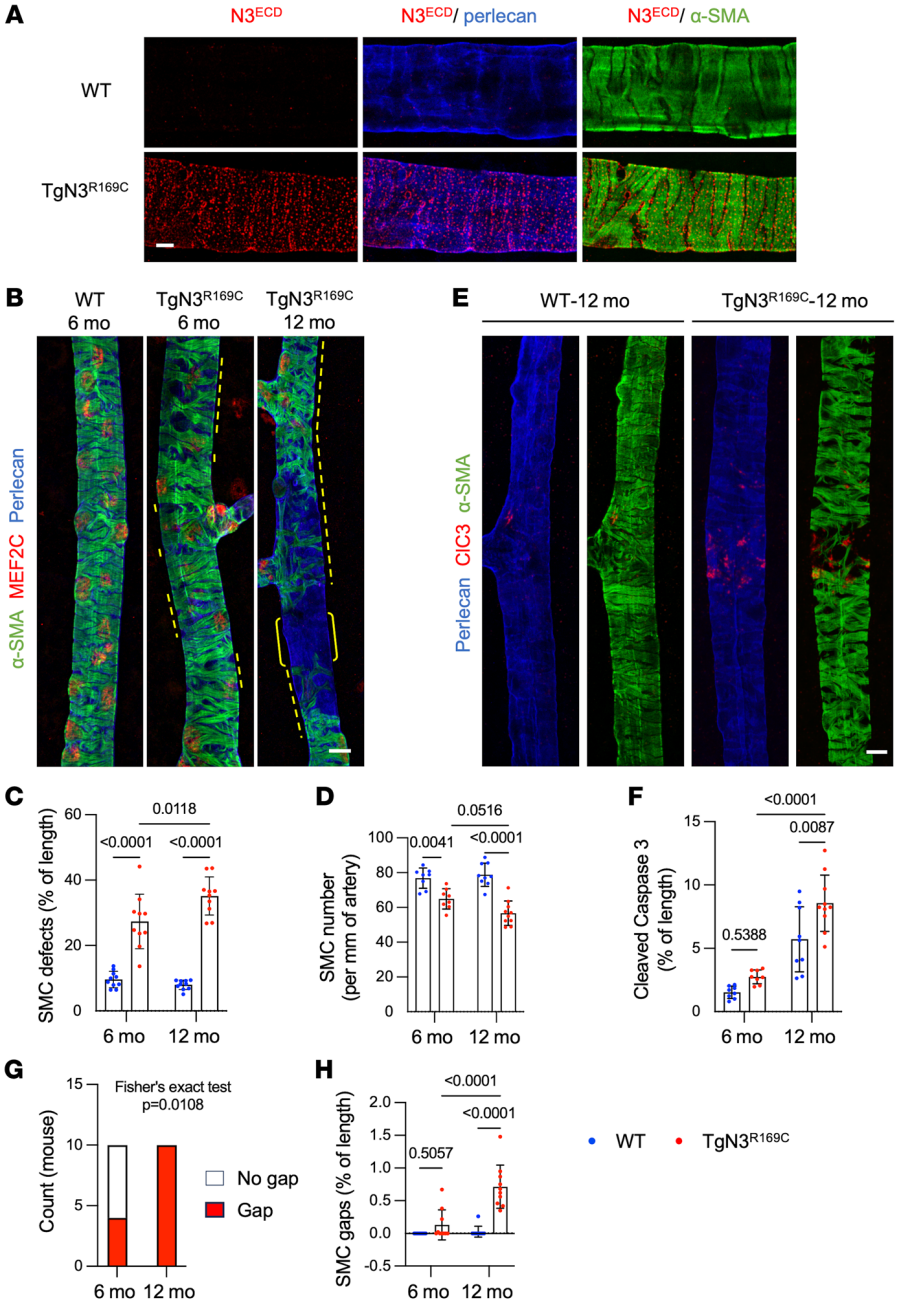
Retinal arteries of Tg*Notch3^R169C^* retinas exhibit progressive degeneration and loss of arterial SMCs. (**A**) Representative confocal images of retinal arteries from WT and Tg*Notch3^R169C^* (TgN3^R169C^) mice, aged 4 months, stained for α-SMA, Notch3^ECD^, and perlecan. (**B**) Representative pictures of retinal arteries from WT and TgN3^R169C^ mice stained for α-SMA, perlecan, and MEF2C. Arteries in mutant mice exhibit a discontinuous SMC coating at 6 months of age onward (dashed yellow lines) that worsens with age, ultimately causing focal SMC gaps (brackets). (**C** and **D**) Quantification of SMC defects (**C**) and SMC numbers (MEF2C nuclei) (**D**) in retinal arteries of WT and TgN3^R169C^ mice at 6 and 12 months of age (*n* = 8–10 mice per group). Data were analyzed by 2-way ANOVA and Tukey’s post hoc test. (**E**) Representative pictures of retinal arteries from WT and TgN3^R169C^ mice, stained for α-SMA, cleaved caspase-3 (ClC3), and perlecan. (**F**) Quantification of cleaved caspase-3 in retinal arteries of WT and TgN3^R169C^ mice at 6 and 12 months of age (*n* = 8–10 mice per group). Data were analyzed by 2-way ANOVA and Tukey’s post hoc test. (**G**) Number of TgN3^R169C^ mice without or with arterial SMC gaps at 6 and 12 months. Data were analyzed by Fisher’s exact test. (**H**) Quantification of SMC gaps in retinal arteries of WT and TgN3^R169C^ mice at 6 and 12 months of age (*n* = 8–10 mice per group). Data were analyzed by 2-way ANOVA and Tukey’s post hoc test. Scale bars: 5 μm (**A**) and 10 μm (**B** and **E**).

**Figure 6:**
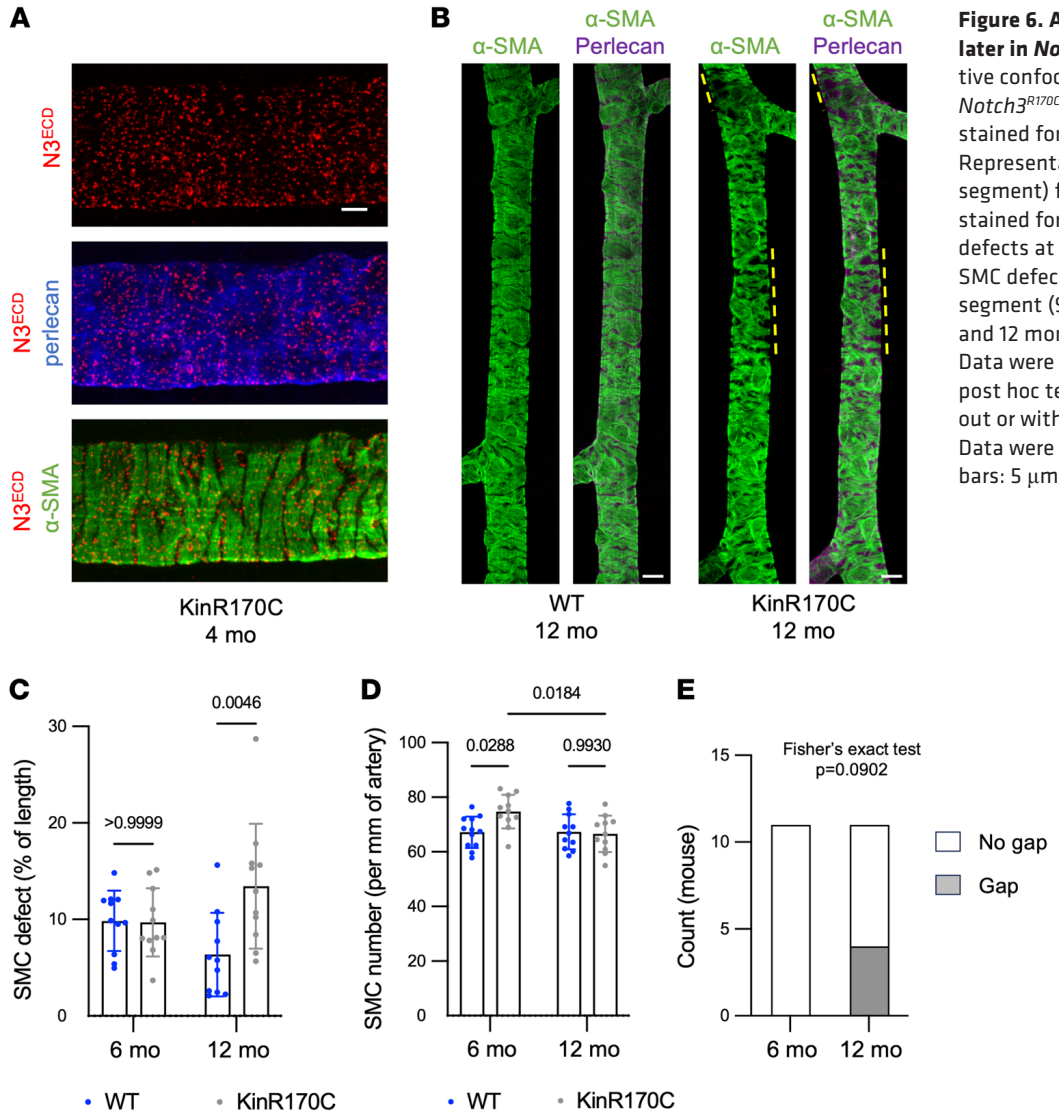
Arterial pathology is milder and appears later in *Notch3^R170C/R170C^* mice. (**A**) Representative confocal images of retinal arteries from *Notch3^R170C/R170C^* (KinR170C) mice aged 4 months, stained for α-SMA, Notch3^ECD^, and perlecan. (**B**) Representative pictures of retinal arteries (distal segment) from *Notch3^+/+^* (WT) and KinR170C mice, stained for α-SMA and perlecan, showing SMC defects at 12 months. (**C** and **D**) Quantification of SMC defects (**C**) and SMC numbers (**D**) in the distal segment (900–1,800 μm) of retinal arteries at 6 and 12 months (*n* = 11 mice per genotype per age). Data were analyzed by 2-way ANOVA and Tukey’s post hoc test. (**E**) Number of KinR170C mice without or with SMC gaps at 6 and 12 months of age. Data were analyzed by Fisher’s exact test. Scale bars: 5 μm (**A**) and 10 μm (**B**).

The 3D morphology of small blood vessels in the brain makes it difficult to assess pathological changes at the cellular level and detect minimal lesions, as might occur in *Notch3^R170C/R170C^* mice. Moreover, a quantitative analysis of arterial defects in the brain is especially burdensome. To overcome these issues, we exploited the stereotyped and planar angioarchitecture of the retina, a developmental extension of the brain that offers unparalleled accessibility for direct analysis and robust quantification of the vascular network at cellular resolution (39, 42). Analyses of the retina are especially relevant given that CADASIL-specific pathologies, including GOM deposits and loss of SMCs, were identified by early studies in retinal vessels of patients with CADASIL (43). Preliminary analyses using flat-mounted retina preparations immunostained for Notch3^ECD^, α-SMA, and perlecan confirmed that retinal arteries of both Tg*Notch3^R169C^* and *Notch3^R170C/R170C^* mice displayed Notch3^ECD^ aggregates (Figure 5A and Figure 6A).

Further analyses of 6-month-old Tg*Notch3^R169C^* mice revealed that retinal arteries of these mice exhibited numerous defects in SMC coverage similar to those observed in brain arteries, whereas such regions were rare in arteries of WT control mice (Figure 5, B and C). To determine whether these defects resulted from a reduction in the number of SMCs, we counted cells with the aid of an antibody against myocyte-specific enhancer factor 2C (MEF2C), a transcription factor that is predominantly expressed in mural cell nuclei (Supplemental Figure 5). The number of MEF2C-positive nuclei was lower in regions with SMC defects compared with regions with normal SMC coverage, and a quantitative analysis confirmed that the number of arterial SMCs was significantly reduced in Tg*Notch3^R169C^* mice (Figure 5, B–D). Moreover, we detected cleaved caspase-3 staining within regions with sparse SMC coating, confirming that the SMCs therein were undergoing apoptosis (Figure 5, E and F). As was the case in the brain, arterial SMC defects were segmental and focal; that is, the burden of defects was unevenly distributed across retinal arteries and within a given artery. Moreover, a distal preference was apparent in Tg*Notch3^R169C^* mice, with more extensive SMC defects observed in the distal region (900–1,800 μm) of arteries compared with the proximal (0–900 μm) region (Supplemental Figure 6). Arterial SMC defects in Tg*Notch3^R169C^* mice worsened with age, becoming significantly more severe at 12 months compared with those at 6 months (Figure 5, B and C). Gaps, which were occasionally detected in Tg*Notch3^R169C^* mice at 6 months, were present in all of these mutant mice at 12 months (Figure 5, G and H).

In *Notch3^R170C/R170C^* mice, SMC coverage of retinal arteries at 6 months of age appeared comparable to that in WT mice, despite a significant increase in the number of arterial SMCs in these mutant mice. At 12 months, SMC defects were detected in the distal region of retinal arteries of *Notch3^R170C/R170C^* mice in association with a return to normal WT numbers of arterial SMCs (Figure 6, B–D). SMC gaps were occasionally observed in 12-month-old *Notch3^R170C/R170C^* mice (Figure 6E).

Collectively, these results indicate that Tg*Notch3^R169C^* and *Notch3^R170C/R170C^* mice exhibit focal and segmental SMC defects and losses in cerebro-retinal arteries, effects that progress with age. They further show that arterial pathology is more severe in Tg*Notch3^R169C^* mice, a feature that is associated with more robust accumulation and aggregation of Notch3^ECD^. A LOF mechanism predicts that, because *Notch3^R170C/R170C^* mice lack normal copies of *Notch3*, arterial defects should be more severe in these mice; thus, our data showing greater effects in Tg*Notch3^R169C^* mice suggest that arterial defects do not result from a reduction in NOTCH3 activity.

### Expression of genes regulated by Notch3 is not increased in brain arteries of TgNotch3^R169C^ mice.

**Figure 7:**
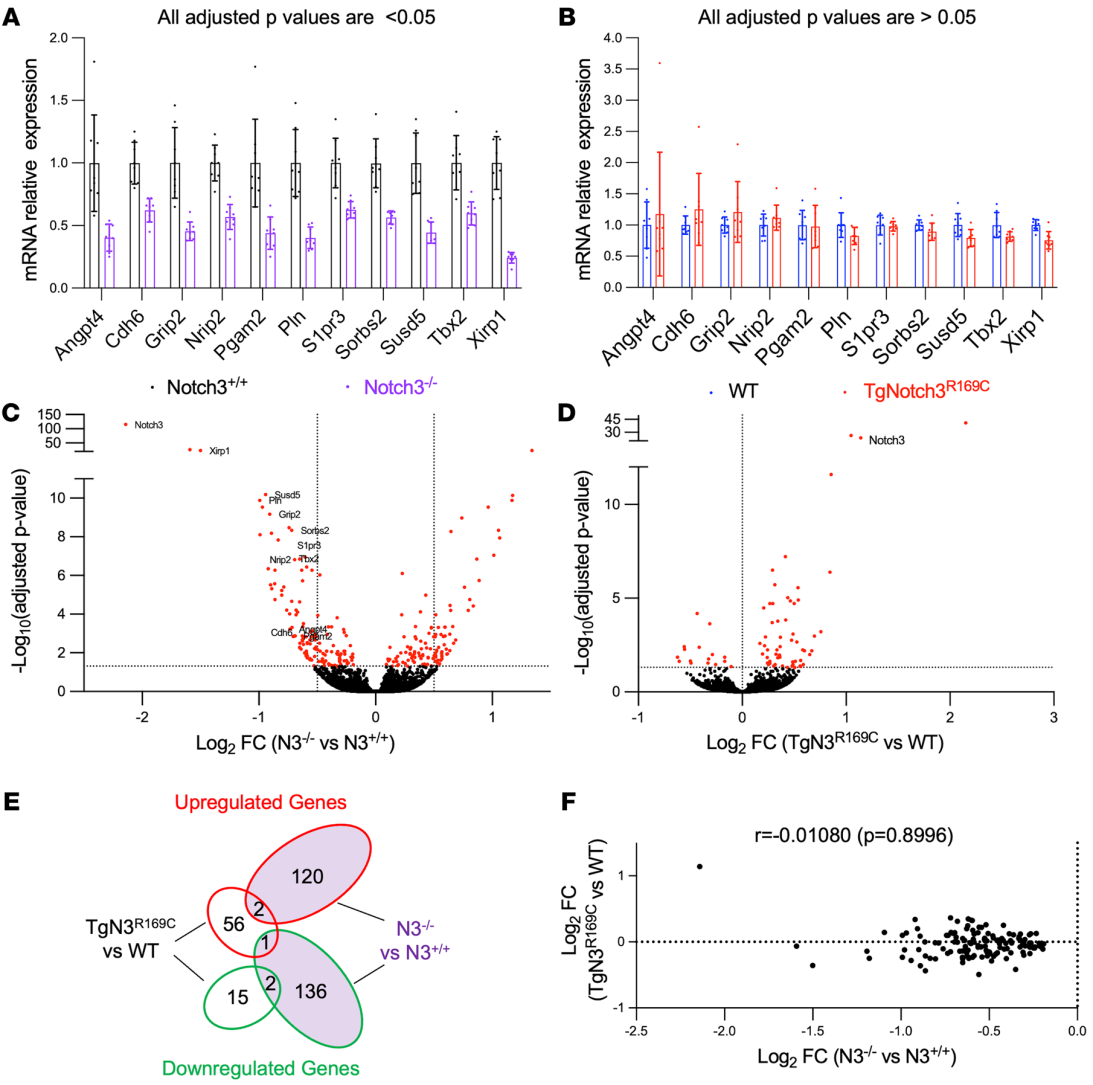
Comparison of gene expression profiles between brain arteries of Tg*Notch3^R169C^* and *Notch3^–/–^* mice. (**A** and **B**) Relative mRNA levels for genes regulated by *Notch3* in brain arteries from *Notch3^–/–^* versus *Notch3^+/+^* mice (**A**) and brain arteries from Tg*Notch3^R169C^* versus WT mice (**B**), measured by RNA sequencing analysis (*n* = 8 mice per genotype). Data are reported as a fold change relative to control mice. Data were analyzed by DESeq2 (Wald test and Benjamini and Hochberg method to correct for multiple testing). (**C** and **D**) Volcano plot representation of RNA sequencing data comparing brain arteries from *Notch3^–/–^* (N3^–/–^) versus *Notch3^+/+^* (N3^+/+^) mice aged 3 months (**C**) and brain arteries from Tg*Notch3^R169C^* (TgN3^R169C^) versus WT mice aged 12 months (**D**) (*n* = 8 mice per genotype). Red symbols indicate DEGs. Data were analyzed by DESeq2 (adjusted *P* value < 0.05). (**E**) Venn diagram showing the overlap of upregulated (red) and downregulated (green) DEGs between TgN3^R169C^ mice (left) and N3^–/–^ mice (right). (**F**) Correlation analysis of fold change (log_2_) values of downregulated genes expressed in N3^–/–^ mice with their fold change (log_2_) values in TgN3^R169C^ mice. Data were analyzed with Spearman’s correlation test.

While our findings above argue against a LOF mechanism, they do not discriminate between increased NOTCH3 activity and a neomorphic effect. To explore the possibility that arterial SMC loss in Tg*Notch3^R169C^* mice might be caused by an increase in NOTCH3 activity, we profiled the transcriptome of brain arteries dissected from Tg*Notch3^R169C^* mice, *Notch3^–/–^* mice, and their WT age-matched littermates using RNA sequencing. We first focused on 11 *Notch3* target genes, which, as expected, were significantly downregulated in brain arteries of *Notch3^–/–^* mice (Figure 7A) (44, 45). None of these 11 genes showed a significant change in Tg*Notch3^R169C^* mice (Figure 7B). Likewise, expression of these 11 genes was unchanged in *Notch3^R170/R170C^* mice (Supplemental Figure 7). Broadening our analysis to the whole genome to ensure that we did not miss a change in the expression of additional *Notch3-*regulated genes in Tg*Notch3^R169C^* mice, we identified 76 differentially expressed genes (DEGs) in Tg*Notch3^R169C^* mice and 261 DEGs in *Notch3^–/–^* mice (Figure 7, C and D). Among these DEGs, only 4 (excluding *Notch3*) were shared by Tg*Notch3^R169C^* and *Notch3^–/–^*, and none of the genes downregulated in *Notch3^–/–^* mice were upregulated in Tg*Notch3^R169C^* or vice versa (Figure 7E). We then focused on significantly downregulated genes in *Notch3^–/–^* mice that have been identified as forming the molecular signature of NOTCH3 activity, assessing the relationship between the log–fold change for each of these genes in *Notch3^–/–^* and their respective log–fold change in Tg*Notch3^R169C^* mice. Again, we found no positive correlation that would be indicative of reduced activity. Importantly, we also found no negative correlation indicative of increased activity (Figure 7F). Taken together, these results suggest that arterial SMC defects in Tg*Notch3^R169C^* mice are not associated with a substantial change in NOTCH3 activity, thus arguing against aberrant NOTCH3 activity and instead supporting a neomorphic effect.

### WT Notch3^ECD^ is incorporated within Notch3^ECD^ aggregates.

**Figure 8:**
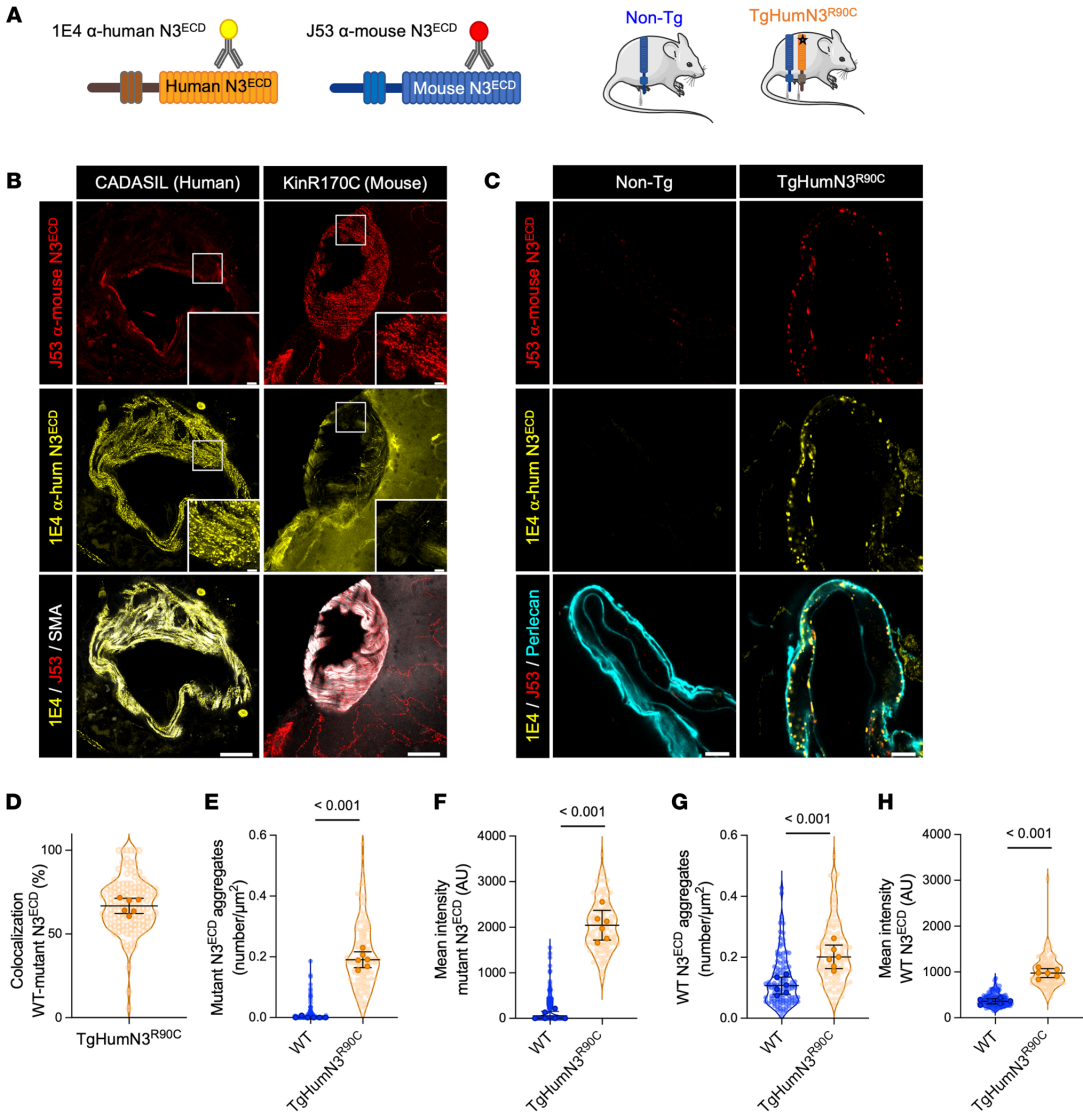
WT Notch3^ECD^ coaggregates with mutant Notch3^ECD^. (**A**) Schematic depiction of anti-Notch3^ECD^ antibodies and the Tg*HumNotch3^R90C^* mouse model. (**B**) Representative confocal images of brain tissue from a *Notch3^R170C/R170C^* (KinR170C) mouse and a CADASIL patient stained with the 1E4 anti–human Notch3^ECD^ (yellow), J53 anti–mouse Notch3^ECD^ (red), and anti–α-SMA (white) antibodies. (**C**) Representative confocal images of brain arteries from WT and Tg*HumNotch3^R90C^* (*n* = 6 per genotype) mice, stained with the 1E4 monoclonal anti–human Notch3^ECD^, J53 anti–mouse Notch3^ECD^, and anti-perlecan (cyan) antibodies. (**D**) Quantification of the colocalization between 1E4- and J53-positive aggregates. Data were analyzed with SODA. (**E**–**H**) Quantification of the number of aggregates containing human mutant Notch3^ECD^ (**E**) or murine WT Notch3^ECD^ (**G**) and the mean intensity of aggregates containing human mutant Notch3^ECD^ (**F**) or murine WT Notch3^ECD^ (**H**) (*n* = 6 per genotype with 20 SMCs per mouse). Shown are violin plots (individual data points represent individual SMCs) and scatter dot plots (individual data points represent individual animals). Data were analyzed with a linear mixed-effects model. Scale bars: 40 μm (**B**) and 5 μm (**C**).

We and others previously showed that overexpression of WT NOTCH3 is not sufficient to cause an abnormal accumulation of Notch3^ECD^ (33, 38, 46). Importantly, prior work has established that, in many neurodegenerative proteinopathies, misfolded proteins that abnormally accumulate act as seeds for aggregation by templating the pathological conversion of their native isoforms, leading to their accumulation (47). In light of this and the findings noted above, we explored the hypothesis that WT NOTCH3 coaggregates with mutant NOTCH3 to participate in the neomorphic effect. To our knowledge, there are no antibodies that specifically recognize the WT or mutant version of NOTCH3. To circumvent this issue, we tested this hypothesis using a transgenic line (Tg*humNotch3^R90C^*) that expresses WT murine NOTCH3 and a human mutant NOTCH3 harboring the CADASIL Arg90Cys mutation (46), employing murine- and human-specific antibodies against Notch3^ECD^ to distinguish between WT (murine) and mutant (human) forms (Figure 8A). As expected, the murine-specific polyclonal antibody (designated J53), raised against rodent NOTCH3, recognized murine Notch3^ECD^ aggregates in *Notch3^R170C/R170C^* mice but did not detect human Notch3^ECD^ aggregates in postmortem brains from CADASIL patients. Conversely, the human-specific monoclonal anti-Notch3^ECD^ antibody (designated 1E4), raised against human NOTCH3, labeled human Notch3^ECD^ aggregates in patients but did not recognize murine Notch3^ECD^ aggregates in *Notch3^R170C/R170C^* mice (Figure 8B). Immunostaining of brain arteries from Tg*humNotch3^R90C^* and WT mice with the human-specific (1E4) antibody showed that human mutant Notch3^ECD^-containing aggregates were present in Tg*humNotch3^R90C^* mice, as expected, and absent in WT mice (Figure 8C). Notably, these aggregates were also labeled by the murine-specific (J53) antibody (Figure 8, C and D), consistent with the idea that WT Notch3^ECD^ is also incorporated into the pathological aggregates. Quantification confirmed that the area, mean fluorescence intensity, and number of both murine-positive aggregates and human Notch3^ECD^-containing aggregates were significantly increased in Tg*humNotch3^R90C^* mice compared with WT mice (Figure 8, E–H).

### Elimination of 1 copy of WT murine Notch3 in TgNotch3^R169C^ mice is sufficient to mitigate Notch3^ECD^ accumulation and arterial pathology.

**Figure 9:**
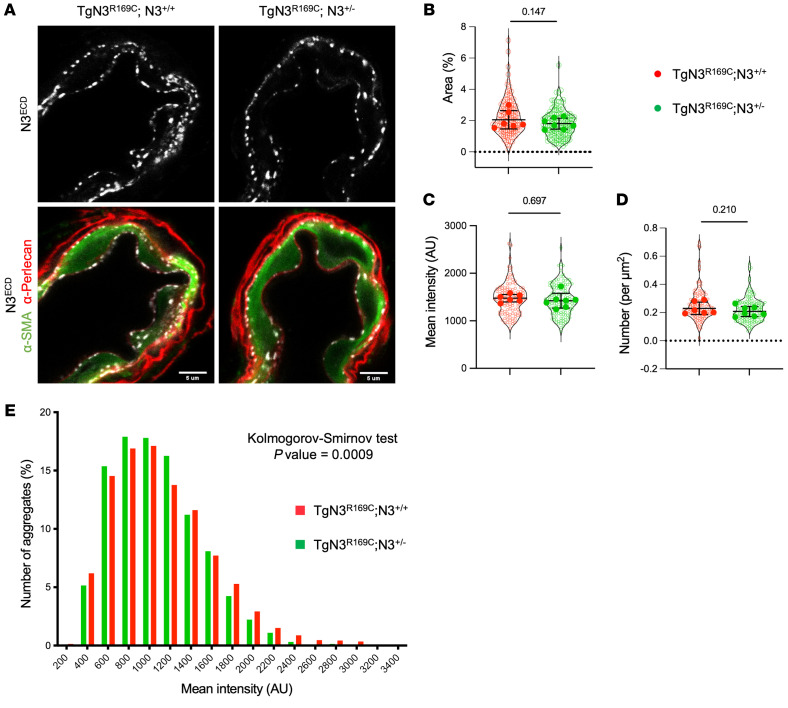
Elimination of one copy of WT *Notch3* attenuates Notch3^ECD^ accumulation in Tg*Notch3^R169C^* mice. (**A**) Representative confocal images of brain arteries from Tg*Notch3^R169C^ Notch3^+/+^* (TgN3^R169C^;N3^+/+^) and Tg*Notch3^R169C^ Notch3^+/–^* (TgN3^R169C^;N3^+/–^) mice stained for α-SMA, perlecan, and Notch3^ECD^. (**B**–**D**) Quantification of the area (**B**), mean intensity (**C**), and number (**D**) of Notch3^ECD^ aggregates in SMCs of TgN3^R169C^;N3^+/+^ and TgN3^R169C^;N3^+/–^ mice (*n* = 6–7 mice per genotype). Shown are violin plots (individual data points represent individual SMCs) and scatter dot plots (individual data points represent individual animals). Data were analyzed with a linear mixed-effects model. (**E**) Intensity distribution of individual Notch3^ECD^ aggregates in TgN3^R169C^;N3^+/+^ and TgN3^R169C^;N3^+/–^ mice. Data were analyzed by Kolmogorov-Smirnov test. Scale bars: 5 μm (**A**).

We next sought to directly establish the centrality of Notch3^ECD^ accumulation in the neomorphic effect and consequent arterial SMC loss by examining how reducing Notch3^ECD^ accumulation impacts arterial pathology. Our demonstration that WT Notch3^ECD^ molecules are present in pathological aggregates predicts that eliminating 1 copy of WT *Notch3* in Tg*Notch3^R169C^* mice would lead to a reduction in Notch3^ECD^ aggregates. To test this, we crossed Tg*Notch3^R169C^* mice with *Notch3*-heterozygous mice (*Notch3^+/–^*), generating Tg*Notch3^R169C^* mice lacking 1 allele of WT *Notch3* (Tg*Notch3^R169C^* *Notch3^+/–^* mice). Quantitative immunofluorescence analyses showed a trend toward a reduction in the number of aggregates in arterial SMCs of Tg*Notch3^R169C^* *Notch3^+/–^* mice compared with Tg*Notch3^R169C^* *Notch3^+/+^* mice (Figure 9A and Supplemental Figure 8). Although this difference did not reach statistical significance using a linear mixed model, employed to assess the interdependence of measurements of distinct SMCs from the same animal (Figure 9, B–D), we found a significant leftward shift in the mean intensity distribution of aggregates in Tg*Notch3^R169C^* *Notch3^+/–^* mice (Figure 9E). These results indicate that the proportion of bright aggregates was lower, and thus the number of Notch3^ECD^ molecules in aggregates was fewer, in mice lacking 1 copy of WT *Notch3* (Tg*Notch3^R169C^* *Notch3^+/–^* mice) than in those expressing both copies (Tg*Notch3^R169C^* *Notch3^+/+^* mice).

**Figure 10:**
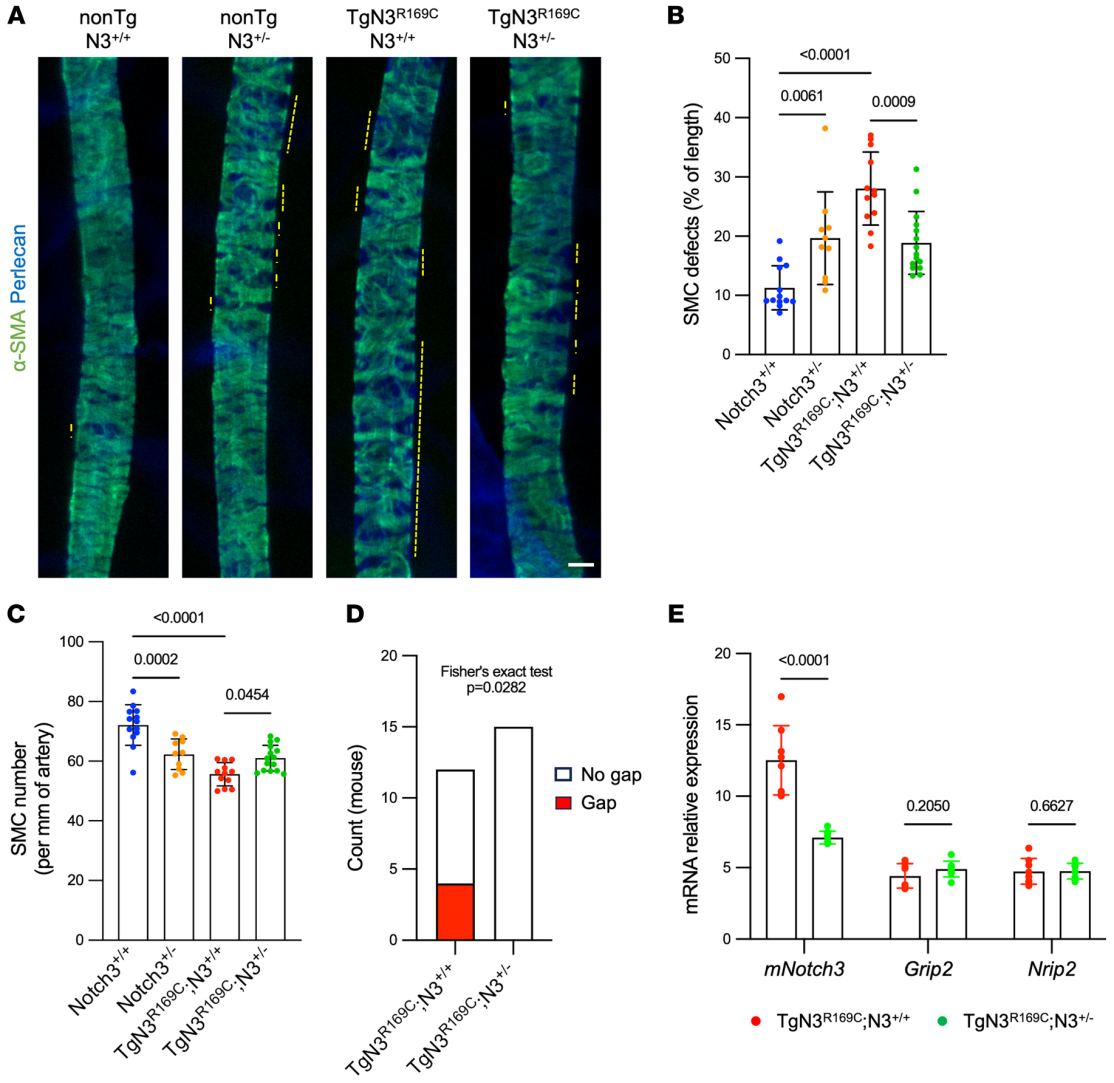
Elimination of one copy of WT *Notch3* mitigates arterial pathology in Tg*Notch3^R169C^* mice. (**A**) Representative images of retinal arteries from non-Tg *Notch3^+/+^* (nonTg;N3^+/+^), non-Tg *Notch3^+/–^* (nonTg;N3^+/–^), Tg*Notch3^R169C^ Notch3^+/+^* (TgN3^R169C^;N3^+/+^), and Tg*Notch3^R169C^ Notch3^+/–^* (TgN3^R169C^;N3^+/–^) mice aged 6 months, stained for α-SMA and perlecan. (**B** and **C**) Quantification of SMC defects (**B**) and SMC numbers (**C**) in retinal arteries from mice aged 6 months (*n* = 10–15 mice per genotype). Data were analyzed by Kruskal-Wallis test followed by Dunn’s post hoc test (**B**) and by 1-way ANOVA followed by Tukey’s post hoc test (**C**). (**D**) Number of TgN3^R169C^;N3^+/+^ and TgN3^R169C^;N3^+/–^ mice without or with SMC gaps in retinal arteries. Data were analyzed by Fisher’s exact test. (**E**) Relative mRNA levels of murine *Notch3*, *Grip2*, and *Nrip2* in TgN3^R169C^;N3^+/+^ and TgN3^R169C^;N3^+/–^ mice, measured by quantitative reverse transcriptase PCR (*n* = 8 per genotype). Data were analyzed by Student’s *t* test. Scale bar: 20 μm (**A**).

We then compared the severity of arterial lesions between Tg*Notch3^R169C^* *Notch3^+/+^* and Tg*Notch3^R169C^* *Notch3^+/–^* mice and their littermate controls at 6 months of age using the retina model. Elimination of 1 copy of *Notch3* in WT mice (*Notch3^+/–^*) at 6 months of age produced arterial SMC defects that were associated with a reduction in the number of arterial SMCs, a finding consistent with the concept that the function of NOTCH receptors is sensitive to gene dosage (2). Strikingly, arterial pathology was attenuated in Tg*Notch3^R169C^* *Notch3^+/–^* mice, which showed significantly fewer arterial SMC defects and a greater number of arterial SMCs compared with Tg*Notch3^R169C^* *Notch3^+/+^* mice (Figure 10, A–C). Moreover, unlike Tg*Notch3^R169C^* *Notch3^+/+^* mice, no Tg*Notch3^R169C^* *Notch3^+/–^* mice exhibited SMC gaps (Figure 10D). To ensure that the amelioration of arterial pathology in Tg*Notch3^R169C^* *Notch3^+/–^* mice did not result from a change in NOTCH3 activity, we quantified brain expression levels of *Grip2* and *Nrip2* — the 2 most sensitive *Notch3* target genes (44) — in addition to murine *Notch3*, by quantitative reverse transcriptase PCR. We found that mRNA levels of *Grip2* and *Nrip2* were indistinguishable between Tg*Notch3^R169C^* *Notch3^+/+^* and Tg*Notch3^R169C^* *Notch3^+/–^* mice, whereas levels of *Notch3* mRNA were decreased by half in Tg*Notch3^R169C^* *Notch3^+/–^* mice, as expected (Figure 10E). Collectively, our results provide compelling evidence that, despite the overt resemblance to loss of NOTCH3 function, CADASIL-associated arterial SMC loss is not driven by a reduction in NOTCH3 activity or by an increase in NOTCH3 activity, but rather by the abnormal accumulation of Notch3^ECD^ — a process in which both mutant NOTCH3 and WT NOTCH3 participate.

## Discussion

CADASIL, the most common heritable cSVD, is caused by highly stereotyped cysteine-altering missense mutations in the NOTCH3 receptor. Two of its defining characteristics are the degeneration and loss of SMCs in small arteries of the central nervous system and abnormal aggregation and accumulation of Notch3^ECD^ around pericytes and SMCs in the brain and retinal microvasculature. Despite the long-standing awareness of these characteristic features, the causal relationship between Notch3^ECD^ accumulation and arterial pathology — and whether altered NOTCH3 activity is involved — has remained a mystery for decades. Research in this field has followed polarized trajectories, with one track favoring an LOF mechanism associated with aberrant NOTCH3 activity, and the other tacking toward the view that abnormal Notch3^ECD^ accumulation results in a neomorphic effect that leads to the gain of a novel function. Because research in the field has pulled in different directions, the development of disease-modifying therapies has lagged. Using a complementary combination of histopathology, imaging, molecular, and genetic approaches, the present study provides strong experimental evidence against the contribution of aberrant NOTCH3 signaling, and instead points to a critical role for Notch3^ECD^ accumulation in CADASIL arterial pathology through coaggregation of both WT and mutant NOTCH3 (Figure 1F).

### Tackling the problem of detecting arterial defects in CADASIL mouse models.

Applying optimized immunostaining and imaging modalities to 200-μm-thick brain sections enabled us to identify arterial defects in the brains of a clinically relevant CADASIL mouse model. These defects were focal and segmental, affecting less than 2% of the arterial bed. This patchy distribution is presumably why such defects have gone unreported to date. In retrospect, it should come as no surprise that 5- to 10-μm-thick sections combined with conventional histological technique would woefully under-sample brain arteries, given this distribution pattern of defects. In addition to slice thickness, our optimization trials highlighted several technical parameters that were key to resolving these limitations, including fixing the brain with paraformaldehyde for a shorter amount of time (1 hour) and performing immunohistochemical incubations at room temperature, which together greatly improved antibody penetration and immunoreactivity. The additional step of postfixation after immunostaining also increased the brightness of the signal and extended its stability up to 18 months. Interestingly, we found that arterial defects equally affected pial arteries running on the surface of the brain, within the cortex, and in deep parts of the brain (Figure 4). Notably, this is despite the fact that, in CADASIL and other cSVDs, brain lesions (white matter lesions and infarcts) predominantly occur in deep brain regions (48). This finding suggests that the heightened vulnerability of deep brain regions is not explained by the topography of arterial pathology but possibly reflects the influence of hemodynamic factors. The flat-mount retina preparation also proved to be instrumental in our ability to characterize arterial defects at the cellular level and robustly quantify these defects in a larger number of animals — and in far less time — than would have been possible in the brain (Figures 5 and 6). Interestingly, arterial defects were seemingly more severe in retinas than brains of mutant mice, possibly reflecting increased sensitivity of detection owing to the planar architecture of the retina preparation, which allows the entire arterial network to be analyzed at unprecedented high resolution. It is also possible that these vascular defects are more severe because the arterial network in the retina is subjected to higher arterial pressure (~80 mmHg) than that in the brain (<50 mmHg). It is notable in this context that hypertension is one of the major risk factors for cSVDs and has been linked to an increased risk of stroke in CADASIL patients (49). Further experiments are needed to determine whether the arterial pathology in CADASIL is exacerbated by increased arterial blood pressure.

### CADASIL — first and foremost an accumulation problem.

The observation that *Notch3*-ablated mice develop arterial SMC loss and exhibit increased susceptibility to stroke has motivated the view that CADASIL pathology is caused by a loss of NOTCH3 function. Our findings strongly refute this hypothesis. If a major driver of arterial pathology were a reduction in NOTCH3 activity as a result of haploinsufficiency — a scenario in which the disease mutation or aggregation of Notch3^ECD^ acts by causing the NOTCH3 receptor to lose its activity — then Tg*Notch3^R169C^* mice, which express the mutation on a normal *Notch3* background, should have a less severe phenotype (or no phenotype at all) compared with *Notch3^R170C/170C^* mice, which express only the mutant receptor. In any case, by no means should Tg*Notch3^R169C^* mice have a more severe phenotype than *Notch3^R170C/170C^* mice (Figures 4–6). In addition, *Notch3^R170C/170C^* mice, like *Notch3*-haploinsufficient (*Notch3^+/–^*) and *Notch3*-ablated (*Notch3^–/–^*) mice, would be expected to exhibit a loss of SMC at 6 months of age, rather than an increased number of SMCs (Figure 6). Conversely, if the presumptive cause were increased NOTCH3 activity, the arterial pathology would be more severe in Tg*Notch3^R169C^* mice than in *Notch3^R170C/170C^* mice, but the expression of NOTCH3 target genes would be increased. Although the first of these predictions is consistent with our findings, our profiling of *Notch3*-regulated genes, performed at the genome-wide scale, does not support the second of these predictions (Figure 7), ruling out this scenario. While an earlier report suggested an increased activity of NOTCH3 in Tg*Notch3^R169C^* mice, this was based on the finding of an increased expression of three NOTCH3 target genes, and in Tg*Notch3^R169C^* model mice on an FBV/N genetic background, not a C57BL/6 background (26). Alternatively, if a major contributor to reduced NOTCH3 activity were a dominant-negative effect — a scenario in which the mutant receptor has lower or no activity and negatively interferes with the normal activity of the WT receptor — the severe arterial pathology of Tg*Notch3^R169C^* mice, which have a mutant-to-WT copy number greater than 1, would be further exacerbated, not attenuated, by the elimination of 1 normal copy of *Notch3* (Figure 10). Taken together, the findings that Tg*Notch3^R169C^* mice, which accumulate more Notch3^ECD^ aggregates, showed more severe arterial defects than *Notch3^R170C/170C^* mice and, more importantly, that a reduction in Notch3^ECD^ accumulation in and of itself was sufficient to attenuate arterial SMC defects, provide strong evidence that Notch3^ECD^ accumulation is the key driver of the arterial pathology. A recent clinical study classified NOTCH3 cysteine-altering variants in 3 different categories (high, medium, and low-risk EGFrs); mutations in high-risk EGFrs (EGFr1–6, 8, 11, and 26) are frequent in CADASIL patients but rare in the general population and lead to a classical CADASIL severe phenotype, whereas mutations in low-risk EGFrs (which mostly include EGFrs in the second half of Notch3^ECD^) are rare in CADASIL patients but frequent in the general population and increasingly encountered in asymptomatic individuals. Interestingly, by study of Notch3^ECD^ accumulation on skin biopsy from mutation carriers and assessment of the effect of mutations on NOTCH3 signaling in vitro, it was found that EGFr risk category was positively associated with Notch3^ECD^ aggregation load, but not with NOTCH3 signaling activity (50, 51), a clinical observation that aligns well with our conclusions.

Our current understanding of how and why Notch3^ECD^ abnormally accumulates in CADASIL is limited. Prior in vitro studies showed that peptides containing WT and mutated EGFr1–5 of NOTCH3 could multimerize to form aggregates (52). Here, we demonstrate that WT Notch3^ECD^ coaggregates with mutant Notch3^ECD^ in arteries (Figure 8) and, more importantly, that WT NOTCH3 participates in the pathogenesis (Figure 10). However, we found that Notch1^ECD^ does not participate in the formation of Notch3^ECD^-containing aggregates (Supplemental Figure 9), although NOTCH1 is also expressed in mural cells of brain vessels and there is some functional redundancy between NOTCH1 and NOTCH3 in these cells (53). Coaggregation of WT and mutant Notch3^ECD^ could reflect a process akin to a thiol-disulfide exchange reaction of a free thiol from a mutant Notch3^ECD^ molecule with a previously disulfide-bonded WT Notch3^ECD^ molecule (54). This would free 1 thiol from a WT Notch3^ECD^ molecule, which could then engage in a reaction with another Notch3^ECD^ molecule, and so on, leading to the formation of WT-mutant Notch3^ECD^ multimers. In addition to its potential therapeutic implications (see below), this result might explain why the phenotype of CADASIL patients carrying a cysteine mutation in the homozygous state is not much more severe than that of patients carrying the same mutation in a heterozygous state (55).

### Trajectories of Notch3^ECD^ accumulation and arterial defects and putative mechanisms underlying the neomorphic effect.

In *Notch3^R170C/R170C^* mice, arterial defects appeared after a significant delay compared with the arterial deposition of Notch3^ECD^. This is reminiscent of the distinct pathogenic phases associated with proteinopathies, such as Alzheimer’s disease, where a first phase characterized by an exponential increase in Aβ accumulation is followed by a second phase during which neurodegeneration occurs (56). The reason for the substantial delay between Notch3^ECD^ accumulation and arterial SMC degeneration is unclear. The deleterious entity may be a late-appearing Notch3^ECD^ species or an indirect Notch3^ECD^ process. Among candidate indirect processes that appear later in the course of the disease and could contribute to arterial pathology is one involving the serine peptidase HTRA1, which we and others have shown accumulates together with Notch3^ECD^ in GOM deposits (8). LOF mutations in HTRA1 cause CARASIL, an inherited recessive cSVD, and dominant-negative HTRA1 mutations cause an autosomal dominant form of cSVD (57–59). Remarkably, brain arteries of CADASIL patients exhibit a protein profile consistent with an HTRA1 loss of function, suggesting that sequestration of HTRA1 together with Notch3^ECD^ in GOM deposits could compromise HTRA1 activity (8). Additional studies are needed to test the hypothesis that arterial SMC loss in CADASIL results from a reduction in HTRA1 activity.

### Implications for therapeutic development.

Three different therapeutic avenues targeting NOTCH3 have been proposed to date: an agonist NOTCH3 antibody to restore NOTCH3 activity, immunotherapy against Notch3^ECD^ to clear Notch3^ECD^ accumulation, and antisense oligonucleotide–mediated exon skipping of specific exons to restore an even number of cysteine residues in the mutant allele (28, 60–62). Our results indicate that, among these approaches, only those aimed at reducing levels of Notch3^ECD^ in arteries are attractive for clinical development. Passive immunization against Notch3^ECD^ showed no significant clearing of Notch3^ECD^ deposits in brain vessels of Tg*Notch3^R169C^* mice, despite almost complete target engagement (61), and active immunotherapy in Tg*Notch3^R182C^* mice showed a significant reduction in Notch3^ECD^ deposition in brain capillaries, but not in brain arteries (62). Proof-of-concept of cysteine correction by exon skipping has been shown in cultured cells, but its ability to reduce NOTCH3 accumulation remains to be assessed (60). Antisense oligonucleotides that induce RNase-mediated degradation of transcripts are ideal candidates for reducing protein expression, and as such hold great promise for the treatment of proteinopathies (63). However, the premise that a loss of NOTCH3 activity could be involved in the pathogenesis has prevented this approach from being explored in CADASIL. Our data indicate that this approach should be highly prioritized. Moreover, our finding that WT NOTCH3 contributes to the disease process suggests that silencing both WT and mutant *Notch3* could be more effective than merely silencing the mutant allele.

### Limitations.

A limitation of our study is its focus on a single mutation. The Arg169Cys mutation is a recurrent mutation in EGFr1–6 that is associated with a highly penetrant and severe disease. We surmise that other mutations in this mutational hotspot could cause small-vessel pathology through the same mechanism. The question is potentially different regarding CADASIL mutations located in and around the ligand-binding domain (EGFr10–11) that are unable to bind ligands and signal and thus behave as LOF mutations. A prior study showed that *Notch3*-KO mice expressing a *Notch3* transgene with the C455R mutation in EGFr11 developed arterial SMC loss that could be prevented by restoring of NOTCH3 signaling (28). However, arterial pathology in this experimental model is not driven by the C455R mutation; instead, it is caused by the lack of endogenous *Notch3* and the inability of the mutant *Notch3* transgene, unlike a WT *Notch3* transgene, to rescue *Notch3* KO–driven SMC loss. Importantly, mutations in EGFr10–11 are still associated with abnormal accumulation of Notch3^ECD^ (16, 64). Moreover, since CADASIL is inherited as a dominant disease, patients carrying such mutations also express 1 normal copy of *NOTCH3*. Therefore, disentangling the contribution of a reduced NOTCH3 activity from Notch3^ECD^ accumulation in CADASIL-driven arterial pathology would require developing mice carrying a cysteine LOF variant in the endogenous *Notch3* locus in the heterozygous state.

### Conclusions.

We are now in a position to detect and quantify CADASIL-related arterial SMC loss in the central nervous system of clinically relevant mouse models. Surmounting this major barrier enabled us to probe the relationship between CADASIL arterial pathology, Notch3^ECD^ accumulation, and NOTCH3 receptor activity — a relationship that has remained enigmatic for more than 20 years. Our results provide experimental evidence that Notch3^ECD^ accumulation, involving both WT and mutant Notch3^ECD^, is a major driver of arterial SMC loss and thus of irreversible vascular dysfunction. Therefore, our data point to NOTCH3-lowering approaches as promising candidate therapeutic strategies.

## Methods

Further information can be found in Supplemental Methods.

### Animals

The Tg*Notch3^R169C^* (33), *Notch3^R170C/R170C^* (26), *Notch3^–/–^* (1), and Tg*HumNotch3^R90C^* (46) mouse lines have been described previously. All 4 lines were backcrossed on a C57BL/6 background at least 6 times. Tg*Notch3^R169C^* mice with half-reduction of *Notch3* (Tg*Notch3^R169C^* *Notch3^+/–^*), together with their control littermates, were generated by crossing of Tg*Notch3^R169C^* and *Notch3^+/–^* mice. Mice were maintained in a specific pathogen–free environment. The transgene in the Tg*Notch3^R169C^* line is integrated on the X chromosome, which is submitted to random X-inactivation in females. However, quantification of *Notch3* transgene mRNA levels in brain arteries showed that the female/male transgene expression ratio was generally greater than 0.5 (mean, 0.867; IQR, 0.809–0.909), indicating that the transgene largely escaped random X-inactivation in females (Supplemental Figure 10). On the basis of these results, we analyzed both Tg*Notch3^R169C^* male and female mice in subsequent studies. Animals were housed under standard conditions (21°C–22°C; 12-hour light/12-hour dark cycle) with food and water provided ad libitum. All animals were randomized and analyzed in a blinded fashion. All experiments were performed on a predetermined number of animals, and no animals were excluded.

### Sex as a biological variable

Our study examined male and female animals, and similar results were found for both sexes.

### Tissue sampling

For the collection of brains or eyes, mice were deeply anesthetized with a mixture of ketamine (180 mg/kg) and xylazine (10 mg/kg) and transcardially perfused with heparin-infused PBS (pH 7.4) or were euthanized by cervical dislocation. The brain was promptly removed from the skull, cut sagittally into 2 halves, flash-frozen in liquid nitrogen, and stored at –80°C until use. For the collection of brain arteries, mice were deeply anesthetized as indicated above and decapitated, after which the brain was rapidly removed from the skull and placed in cold PBS. After incubation for 30 minutes on ice, brain arteries were dissected under a stereomicroscope, then used immediately (immunolabeling) or flash-frozen in liquid nitrogen and stored at –80°C until use (RNA or protein extraction). Eyes were fixed by incubation in 4% paraformaldehyde (PFA) for 2 hours at 4°C, then rinsed twice in PBS containing 0.02% sodium azide (PBS-A) and stored at 4°C for up to 1 week before use. For immunostaining, the retina was dissected under a stereomicroscope and used immediately.

### Human tissues

Postmortem brain samples (frontal lobes) from 3 CADASIL patients with a cysteine NOTCH3 mutation were used.

### Immunostaining

Primary antibodies against the following proteins (species, dilution, catalog number, supplier) were used: FITC-conjugated α-SMA (mouse, 1:1,000, clone 1A4, F3777, Sigma-Aldrich), cleaved caspase-3 (Asp175) (rabbit, 1:1,000, 9661, Cell Signaling), elastin (rabbit, 1:1,000; provided by R. Mecham, Washington University School of Medicine, St. Louis, Missouri, USA), MEF2C (rabbit, 1:1,000, clone D80C1, 5030, Cell Signaling), Notch3^ECD^ (J53: rabbit, 1:16,000 or 1:32,000, ref. 61; 1E4: mouse, 1:200, ref. 6), heparan sulfate proteoglycan (perlecan) (rat, 1:2,000 or 1:3,000, clone A7L6, MAB1948P, Millipore). The following secondary antibodies were used: Alexa Fluor 594–conjugated donkey anti-rabbit, Alexa Fluor 647–conjugated donkey anti-rat, Alexa Fluor 488–conjugated donkey anti-mouse, Alexa Fluor 647–conjugated donkey anti-rabbit (1:500, Life Technologies), and Alexa Fluor 568–conjugated donkey anti-rat (1:500, ab175475, Abcam).

Frozen brain hemispheres (mouse) or brain samples (human) were thawed in 4% PFA for 1 hour at 4°C on a rotating shaker, rinsed twice in PBS, and cut into 200-μm-thick coronal sections using a vibratome (VT 1200, Leica) before immunostaining. Dissected retinas or brain sections were blocked in PBS containing 10% FBS and 0.2%–0.5% Triton X-100 at room temperature (RT) for 2 hours, and incubated first with primary antibodies, diluted in PBS containing 1% FBS and 0.2% Triton X-100 for 24 hours at RT, and then with secondary antibodies for 4 hours at RT in the same buffer. Brain slices were postfixed in 4% PFA at RT for 15 minutes, counterstained with 1 μg/mL DAPI to label nuclei, and then mounted onto glass slides with Dako Fluorescence Mounting Medium (Agilent Technologies) under a coverslip. Retinas were postfixed and coverslip-mounted using the same procedure.

Brain arteries (middle cerebral arteries) were dissected under a stereomicroscope and fixed in a solution containing 3% glyoxal (Sigma-Aldrich), 20% ethanol, and 0.75% acetic acid (buffered at pH 4; Figures 2 and 9), or 4% PFA (Figure 8), for 1 hour on ice, then rinsed twice in PBS. Arteries were blocked in PBS containing 10% FBS and 0.1% Triton X-100 at RT for 2 hours, then incubated first with primary antibodies, diluted in PBS containing 1% FBS and 0.1% Triton X-100 for 24 hours at RT, and then with secondary antibodies overnight at RT in the same buffer. Arteries were then postfixed in 4% PFA at RT for 15 minutes and incubated with DAPI (1 μg/mL) for 5 minutes, after which individual arteries were embedded in an upright position in a cryomold containing 3% low-gelling-temperature agarose (Sigma-Aldrich; dissolved in PBS) under a stereomicroscope for subsequent cross-sectioning. The agarose block was cut into 200-μm-thick sections using a vibratome, and then sections were coverslip-mounted onto glass slides with Dako Fluorescence Mounting Medium and Grace Bio-Labs SecureSeal imaging spacers (Sigma-Aldrich).

### Imaging

Brain slices and retinas were imaged with a Zeiss Axio Observer wide-field epifluorescence microscope equipped with an *xyz* motorized stage, a multicolor LED light source (Colibri 7, Zeiss), and a 16-bit ORCA-Flash 4.0 OLT+ digital CMOS camera (C11440, Hamamatsu). Tiling mode and post-acquisition stitching were used to capture and image the entire retina or a whole brain section. Images were repeatedly captured using the same exposure times and LED light power. Higher-resolution images of retinas or brain slices were acquired with a TCS SP5 or an SP8 laser-scanning confocal microscope (Leica Microsystems) using ×63 (NA 1.4, oil immersion), ×40 (NA 1.3, oil immersion), or ×20 (NA 0.75, oil/glycerol immersion) objectives. Brain arteries were imaged with a TCS SP5 laser-scanning confocal microscope using a ×63 (NA 1.4) oil immersion objective.

### Quantitative analysis

All quantifications were performed using Icy (https://icy.bioimageanalysis.org/) or ImageJ (https://imagej.nih.gov/ij/) open-source software, and all procedures were performed with prefixed parameters under strictly blinded conditions.

#### Vascular pathology in the retina.

Quantification was performed on flat-mounted retinas labeled for α-SMA, MEF2C, and perlecan (1 retina per mouse). Epifluorescence images were obtained using a ×20 objective (NA 0.4, 1,800 × 1,800 pixels per image, 0.325 μm/pixel, 16 bits), and a seamless mosaic image of the whole retina was reconstructed.

#### Arterial SMC defects and gaps.

Arteries within a 1.8 mm radius centered on the optic nerve were visually inspected for gaps or defects in α-SMA labeling. Arterial segments were further divided in proximal (0–0.9 mm) and distal (0.9–1.8 mm) regions. The number of SMC gaps, the lengths of arterial segments with SMC defects or SMC gaps, and the total length of arterial segments were measured. Results are expressed as the percentage of the arterial bed containing gaps or defects.

#### Number of arterial SMCs.

Arteries (proximal and distal segments) within a 1.8 mm radius centered on the optic nerve were manually segmented on the perlecan channel. Care was taken to count only MEF2C-positive nuclei that were fully contained within the perlecan channel. Data are reported as the number of arterial SMCs per millimeter of arteries.

#### Vascular pathology in the brain.

Quantification was performed on coronal brain sections labeled for α-SMA, elastin, and perlecan (4 sections per mouse, 2 anterior sections starting at bregma 1.10 mm and 2 posterior sections starting at bregma –1.06 mm). Epifluorescence images were obtained using a ×5 objective (NA 0.16, 2,048 × 2,048 pixels per image, 1.3 μm/pixel, 16 bits), and a seamless mosaic image of the whole-brain slice was reconstructed. Arteries/arterioles — defined as vessels stained by α-SMA, elastin, and perlecan — were visually inspected for discontinuity in α-SMA staining. A discontinuous pattern of α-SMA staining was classified as an “SMC gap” if it spanned the entire vessel width and an “SMC defect” if not. The number of SMC gaps, the lengths of arterial segments with SMC defects, and the total length of arterial segments were measured. Results are expressed as the percentage of the arterial bed containing gaps or defects.

#### Notch3^ECD^ staining in brain arterial SMCs.

Quantification was performed on brain arteries labeled for α-SMA, Notch3^ECD^, and perlecan. Images were acquired with a TCS SP5 confocal microscope using a ×63 (NA 1.4, 512 × 512 pixels per image, 74 nm/pixel, 12 bits) oil immersion objective. SMCs, identified as α-SMA–positive cells surrounded by a perlecan-positive membrane, were segmented using the “active contour” Icy plug-in, then dilated by 2 pixels. Notch3^ECD^ staining was segmented within each SMC using the Spot Detector Icy plug-in, which detects and counts particles based on wavelet, with scale and sensitivity set to 2 and 100, respectively. An intensity-based threshold (95th percentile of signal intensity) was applied to Notch3^ECD^ spots based on the distribution of particle intensities in SMCs from *Notch3^–/–^* mice processed in the same batch. A fixed number (*n* = 20) of randomly chosen SMCs was analyzed per mouse. Results are expressed as the mean percentage of Notch3^ECD^ staining area and the mean intensity of Notch3^ECD^ staining per SMC and per mouse. Notch3^ECD^ aggregates were specifically detected by setting the sensitivity in Spot Detector to 20. Results are expressed as the mean percentage of Notch3^ECD^ aggregate area, the mean intensity of Notch3^ECD^ aggregates, and the mean number of Notch3^ECD^ aggregates per SMC and per mouse. Also shown are the size and mean intensity distribution of aggregates per genotype. Colocalization was quantified using Statistical Object Distance Analysis (SODA) (65).

### Statistics

Unless specified otherwise, graphs in figures display individual values and mean ± SD. Analyses were performed using GraphPad Prism version 10.1.1. Data for experiments with more than 8 animals per group were subjected to D’Agostino and Pearson normality tests. The significance of differences between 2 groups was determined using unpaired, 2-tailed Student’s *t* test or, in cases in which experiments did not satisfy normality tests, 2-tailed Mann-Whitney test. Differences among more than 2 groups were compared using 1-way ANOVA followed by Tukey’s post hoc test (normally distributed data) or Kruskal-Wallis test followed by Dunn’s post hoc test (non-normally distributed data). Since the Mann-Whitney test underperforms on small sample sizes, and variance calculations are meaningless for smaller samples, experiments with groups of 8 or fewer distinct samples were analyzed using unpaired, 2-tailed Student’s *t* test or 1-way ANOVA with Tukey’s post hoc test correction for multiple comparisons. Two-way ANOVA followed by Šidák’s or Tukey’s post hoc test was used for analysis of 2 independent variables. Fisher’s exact test of contingency was used to compare proportions between 2 groups. For cases in which multiple measurements were obtained from the same animal, we used a linear mixed-effects model to take into account data dependency (JASP open-source software; https://jasp-stats.org/). A Kolmogorov-Smirnov test was used to compare cumulative distributions. *P* less than 0.05 was considered as statistically significant. Differentially expressed genes were determined using the R 3.3.2 package DESeq2 (version 1.16.1 Bioconductor library) (66). Briefly, differential expression between 2 groups was analyzed using the Wald test, and the *P* values attained by the Wald test were corrected for multiple testing using the Benjamini and Hochberg method. Adjusted *P* less than 0.05 was considered as statistically significant.

### Study approval

Human samples were stored and handled in accordance with the French bioethics laws, and the study was approved by the Institutional Review Board of INSERM. All mouse experiments were approved by our local Institutional Animal Care and Use Committees (CEEA Paris Descartes no. 34) and by the French government (Ministère de l’Enseignement Supérieur et de la Recherche).

### Data availability

RNA sequencing data generated in this study were deposited in the NCBI Gene Expression Omnibus database under accession code GSE242794. Other results are reported in the Supporting Data Values file.

## Author contributions

ND, assisted by VDD, CN, and VD, performed histopathological analyses, analyzed the data, prepared the figures, and assisted in the writing of the manuscript. FG studied the expression of Notch3^ECD^ in brain arteries, analyzed the data, and drafted the figures. CK and CMR, assisted by VDD and VD, performed and analyzed transcriptomic data. LD designed the epifluorescence microscope setup and assisted with confocal microscopy imaging, image analysis, and quantifications. DHE and MTN provided intellectual input and edited the manuscript. AJ designed the study, supervised the research, analyzed data, and wrote the manuscript.

## Supplementary Material

Supplemental data

Unedited blot and gel images

Supporting data values

## Version 1 02/22/2024

In-Press Preview

## Version 2 04/15/2024

Electronic publication
