# Supplementary material for: Protein aggregates containing wild-type and mutant NOTCH3 are major drivers of arterial pathology in CADASIL
Source: J Clin Invest. 2024 Feb 22;134(8):e175789. doi: 10.1172/JCI175789 (PMC11014667; doi:10.1172/JCI175789)
Supplement: Supplemental data [file jci-134-175789-s208.pdf]

Supplementary material

Title: Protein aggregates containing wild-type and mutant NOTCH3 are major drivers of arterial pathology in CADASIL

Authors

Nicolas Dupré [1], Florian Gueniot [1], Valérie Domenga-Denier [1], Virginie Dubosclard [1], Christelle Nilles [1], David Hill-Eubanks [2], Christelle Morgenthaler-Roth [3], Mark T. Nelson [2,4], Céline Keime [3], Lydia Danglot [1], Anne Joutel [1,2,5].

Affiliations

1- Université Paris Cité, Institute of Psychiatry and Neuroscience of Paris (IPNP), INSERM U1266, 75014 Paris, France

2- Department of Pharmacology, College of Medicine, University of Vermont, Burlington, VT, USA

3- Institut de Génétique et de Biologie Moléculaire et Cellulaire, CNRS UMR 7104, INSERM U1258, Université de Strasbourg, Illkirch, France

4- Division of Cardiovascular Sciences, University of Manchester, Manchester, UK

5- GHU-Paris Psychiatrie et Neurosciences, Hôpital Sainte Anne, F-75014 Paris, France

Corresponding author

Anne Joutel, Inserm U1266, Institute of Psychiatry and Neurosciences of Paris, 102-108 rue de la Santé, 75014 Paris, France. Email: anne.joutel@inserm.fr. Tel: +33 1 40 78 92 96

Supplementary methods

Protein extraction and Immunoblot analysis

Brain arteries from 12-month-old Tg*Notch3*^{R169C} and age-matched WT littermates were lysed in RIPA buffer (150mM NaCl, 50 mM Tris-HCL, 1% NP-40, 0.1% SDS, 0.5% sodium deoxycholate supplemented with protease inhibitors (Halt™ Protease inhibitor Single use cocktail; Thermo Scientific) at 4°C and centrifuged at 21,000 g for 30 min at 4°C (n= 3 samples, each sample made from 2 mice per genotype). The supernatant was saved (RIPA soluble) and mixed with SDS-Laemmli buffer (4% SDS, 125 mM Tris-HCl, pH 6.8, 20% glycerol) with 50 mM DTT; the resultant insoluble pellet (RIPA insoluble) was extracted 36 hrs in SDS-Laemmli buffer with 50 mM DTT. Protein extracts were heated for 5 min at 95°C, electrophoresed on a 6% Tris-Glycine SDS-PAGE and transferred to a nitrocellulose membrane. The membrane was stained with SYPRO Ruby (ThermoFisher Scientific) to detect total proteins prior to immunoblotting. The following primary antibodies were used: mouse monoclonal anti-Notch3^{ECD} (a biotinylated version of clone 5E1, dilution 1:5000)(1), sheep polyclonal anti-mouse Notch1^{ECD} (AF 5267, Bio-Techne, dilution 1:250). Blots were incubated with the primary antibody at 4°C overnight followed by anti-sheep immunoglobulins (Bio-Techne) or streptavidin (Cell Signaling Technology) conjugated with horseradish peroxydase and ECL detection (Super Signal™ West Femto maximum sensitivity and Super Signal™ West Pico PLUS chemiluminescent substrates, Thermo Scientific). Densitometric quantification of band intensity was performed using ImageJ. Results are presented as total protein–normalized relative expression levels

Reverse transcription and quantitative polymerase chain reaction (RT-qPCR)

Brain arteries (n = 8 mice/genotype) were homogenized on ice in lysis buffer using a polytron homogenizer, and total RNA was extracted following the manufacturer's protocol (RNeasy MicroKit, Quiagen). cDNA was synthesized from total RNA using M-MLV reverse transcriptase (ThermoFisher Scientific). Quantitative PCR was performed on a CFX Connect Real-Time PCR detection system (Bio-Rad, Oxford, UK) using SYBR Green PCR master mix (Bio-Rad). The primer pairs used are indicated in our previous publication (2). Results are presented as β-actin–normalized relative expression levels.

Bulk RNA sequencing

Brain arteries from 12-month-old Tg*Notch3*^{R169C} and age-matched WT littermates, 12-month-old *Notch3*$^{R170C/R170C}$ and age-matched littermates as well as 3-month-old *Notch3*$^{-/-}$ and age-matched *Notch3*$^{+/+}$ mice (n = 8 mice per genotype and age) were homogenized on ice in lysis buffer using a polytron homogenizer, and

total RNA was extracted following the manufacturer's protocol (RNeasy MicroKit, Quiagen). Sample quality was assessed using an Agilent 2100 bioanalyzer (Agilent Technologies). RNA-Seq libraries were generated from 150 ng of total RNA using Illumina Stranded mRNA Prep, Ligation kit and IDT for Illumina RNA UD Indexes Ligation (Illumina) according to the manufacturer's instructions. Following polyA selection, mRNAs were fragmented at 94°C for 8 minutes. DNA libraries were amplified using 13 cycles of PCR. Surplus PCR primers were further removed by two successive purifications using SPRIselect beads (Beckman-Coulter). The final libraries were checked for quality and quantified using a Bioanalyzer 2100 system (Agilent Technologies). Libraries were then sequenced on an Illumina HiSeq4000 sequencer as single-end 50-base reads. Image analysis and base calling were performed using RTA 2.7.7 and bcl2fastq 2.20.0.422. Adapter, polyA and low-quality sequences (Phred quality score < 20) were removed by preprocessing using cutadapt (3) version 1.10. Reads shorter than 40 bases and those corresponding to rRNA, mapped using bowtie version 2.2.8 (4), were discarded for further analysis. Remaining reads were then mapped onto the mm39 assembly of mouse genome using STAR version 2.5.3a (5). Gene expression was quantified using htseq-count (6) version 0.6.1p1 and gene annotations from Ensembl release 106.

References

1. Joutel A, et al. The ectodomain of the Notch3 receptor accumulates within the cerebrovasculature of CADASIL patients. *J Clin Invest*. 2000;105(5):597–605.

2. Ratelade J, et al. Reducing Hypermuscularization of the Transitional Segment Between Arterioles and Capillaries Protects Against Spontaneous Intracerebral Hemorrhage. *Circulation*. 2020;141(25):2078–2094.

3. Martin M. Cutadapt removes adapter sequences from high-throughput sequencing reads. *EMBnet.journal*. 2011;17(1):10.

4. Langmead B, Salzberg SL. Fast gapped-read alignment with Bowtie 2. *Nat Methods*. 2012;9(4):357–359.

5. Dobin A, et al. STAR: ultrafast universal RNA-seq aligner. *Bioinforma Oxf Engl*. 2013;29(1):15–21.

6. Anders S, Pyl PT, Huber W. HTSeq--a Python framework to work with high-throughput sequencing data. *Bioinforma Oxf Engl*. 2015;31(2):166–169.

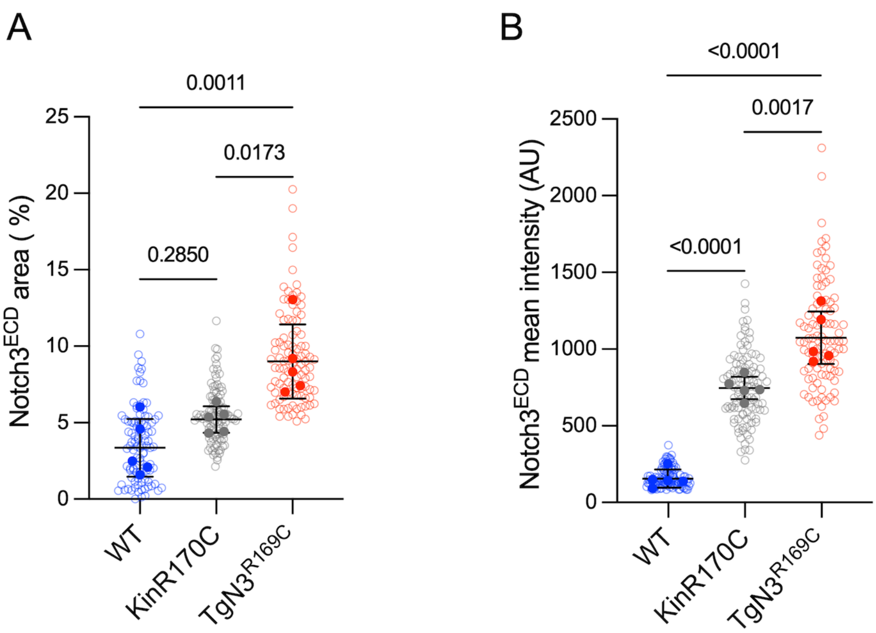

Supplemental Figure 1: Quantification of Notch3^{ECD} staining in brain arterial SMCs using a non-stringent protocol. A, B. Quantification of the mean area (**A**) and mean intensity (**B**) of Notch3^{ECD} labeling in brain arterial SMCs of WT, *Notch3*$^{R170C/R170C}$ (KinR170C), and Tg*N3*^{R169C} (TgN3^{R169C}) mice aged 4 months (n= 5 mice/genotype and 20 SMCs per mouse). Shown are scatter dot plots, where empty circles represent individual SMCs and full symbols represent individual animals. Data from individual mice were analyzed by a one-way ANOVA and Tukey's post-hoc test.

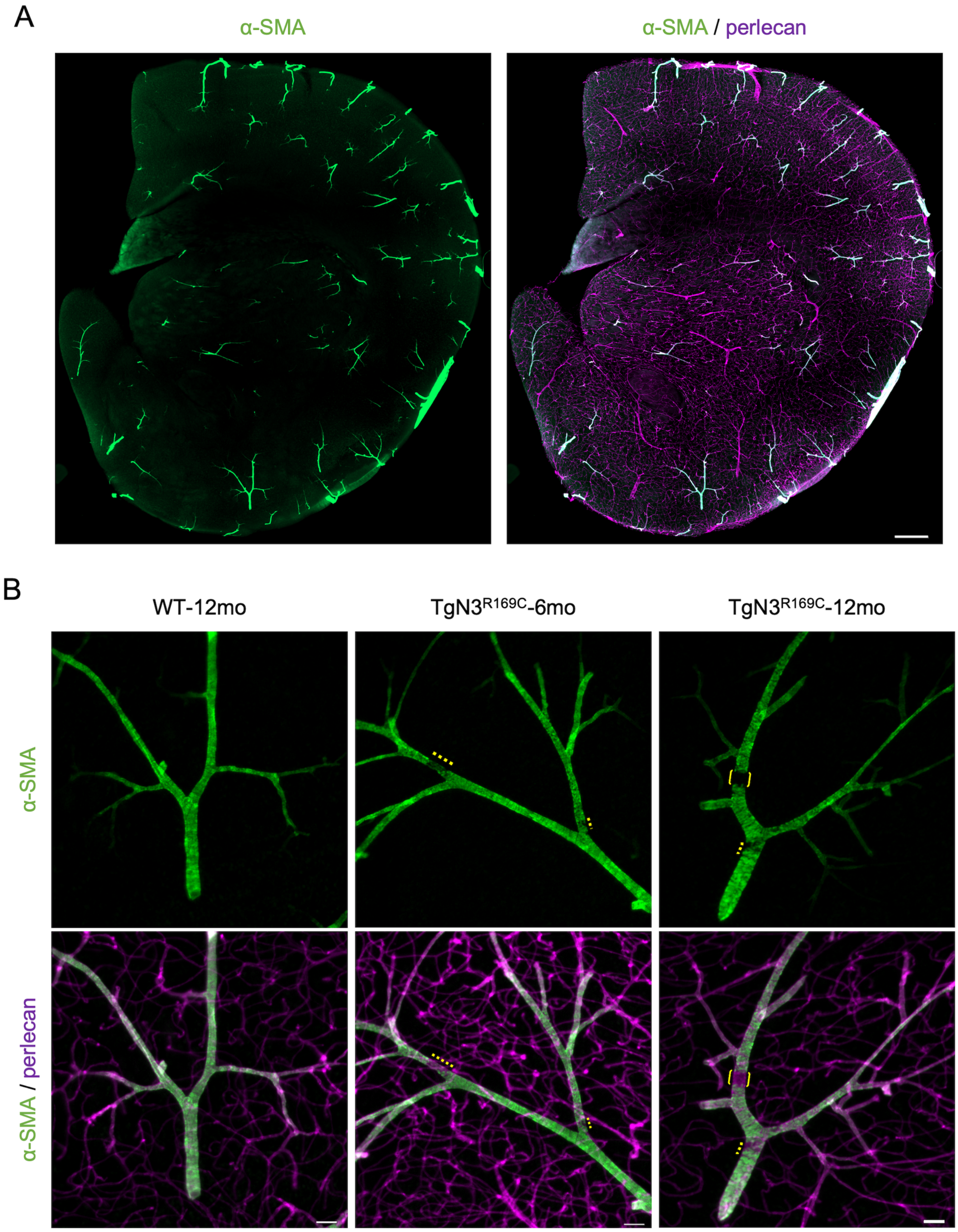

Supplemental Figure 2: Imaging of thick brain slices with a widefield epifluorescence microscope enables the detection of arterial defects in mutant mice. Images of 200-µm-thick brain coronal sections stained for α-SMA, elastin, and perlecan, acquired with a Zeiss Axio Observer microscope and a 5X objective (0.16 numerical aperture, 1.3 × 1.3 µm^2 pixel size). (**A**) Representative mosaic images of an entire brain section. (**B**) Magnified images of representative brain arteries from a WT mouse aged 12 months and Tg*N3^{R169C}* mice aged 6 or 12 months. The resolution and acquisition mode allow the detection of focal SMC defects (yellow dotted lines) and gaps (brackets) in mutant brain arteries. Scale bars: 500 µm (**A**) and 50 µm (**B**).

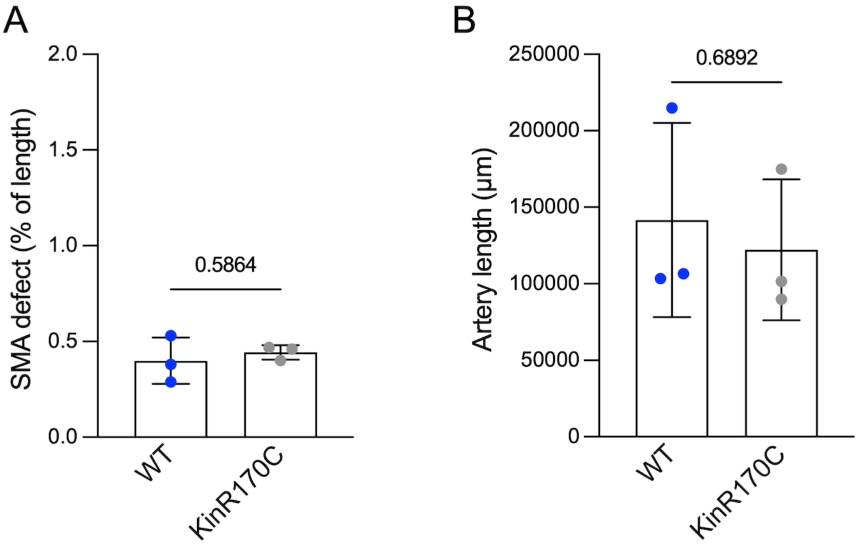

Supplemental Figure 3: The integrity of brain arteries from *Notch3*$^{R170C/R170C}$ is preserved until 22 months of age. A. Quantification of SMC defects in Notch3$^{+/+}$ (WT) and *Notch3*$^{R170C/R170C}$ (KinR170C) mice aged 22 months in brain arteries (n=3 mice/genotype). Data were analyzed by a student's t-test. **B.** Quantification of arterial length in the brain of WT and KinR170C mice aged 22 months. Data were analyzed by Student's t-test.

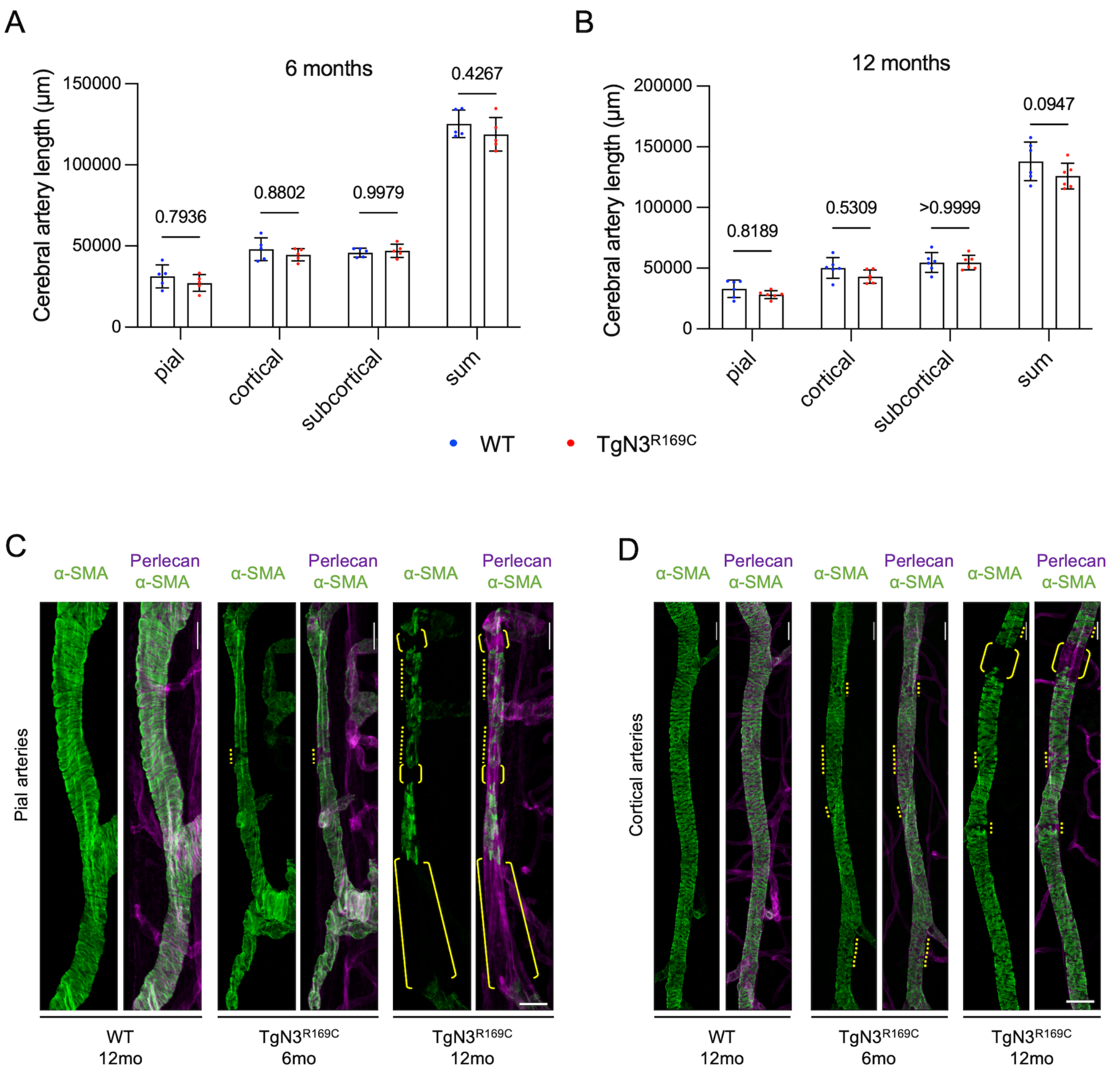

Supplemental Figure 4: SMC defects in Tg*Notch3*^{R169C} mice are also detectable in pial and cortical arteries. A. Quantification of global and region-specific arterial length in the brains of WT and Tg*N3*^{R169C} mice at 6 (**A**) and 12 (**B**) months (n = 5–6 mice/genotype; 4 brain slices per mouse). Data were analyzed by 2-way ANOVA and Sidak's post-hoc test. **C, D.** Confocal images of 200-µm-thick brain sections stained for α-SMA, elastin, and perlecan. Shown are representative pial (**C**) and cortical (**D**) brain arteries from WT and TgN3^{R169C} mice. Arteries in TgN3^{R169C} mice aged 6 months and older exhibit SMC defects (yellow dotted lines) as well as α-SMA gaps (brackets). Scale bars: 20 µm (**C, D**).

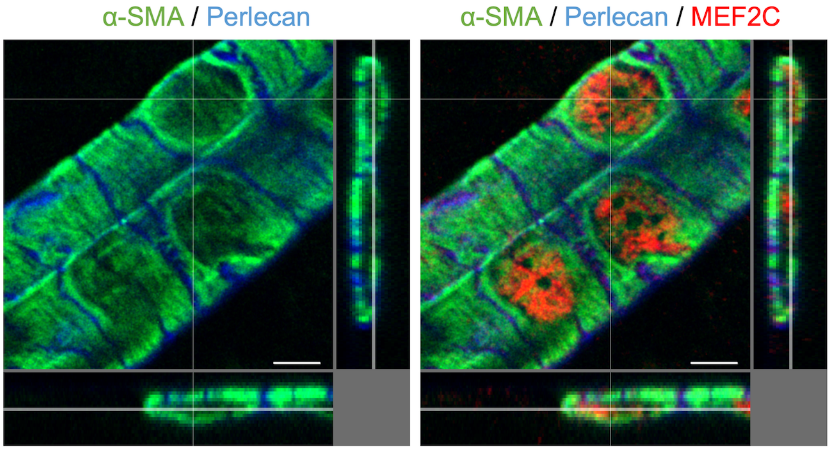

Supplemental Figure 5: Anti-MEF2C antibody stains the nucleus of mural cells and enables their counting. Representative confocal images of a retinal artery stained for α-SMA, MEF2C and perlecan, with orthogonal views demonstrating that MEF2C-positive nuclei are surrounded by α-SMA staining.

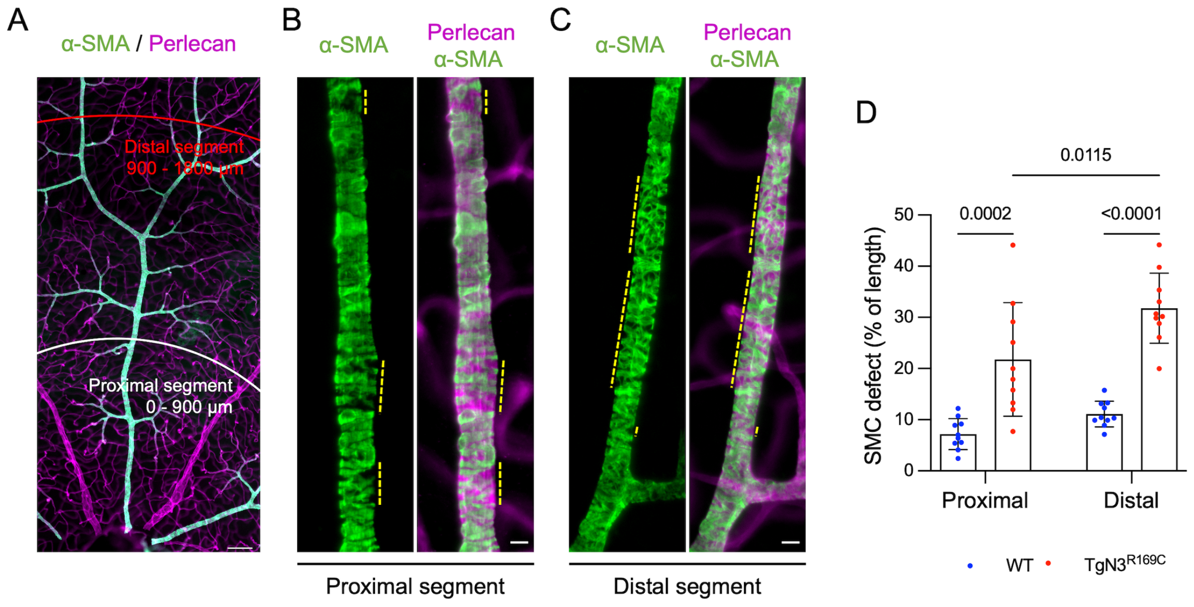

Supplemental Figure 6: SMC defects in Tg*Notch3^{R169C}* mice are focal and more severe in the distal part of retinal arteries. A. Representative image of a retina stained for α-SMA and perlecan illustrating the proximal and distal segments of a retinal artery, defined as those within a 0–900 µm and 900-1,800 µm radius, respectively, centered on the retina optic nerve. **B, C.** Representative images of proximal (**B**) and distal (**C**) segments of an artery stained for α-SMA and perlecan from a 6-month-old Tg*Notch3^{R169C}* mouse illustrating that SMC defects (yellow dotted lines) are focal (i.e., surrounded by normal appearing SMC coverage and more pronounced in the distal segment). **D.** Quantification of SMC defects in proximal and distal arterial segments in 6-month-old WT and Tg*Notch3^{R169C}* (TgN3^{R169C}) mice (n = 10 mice/genotype). Data were analyzed by 2-way ANOVA and Tukey's post-hoc test. Scale bars: 100 µm (**A**) and 10 µm (**B, C**)

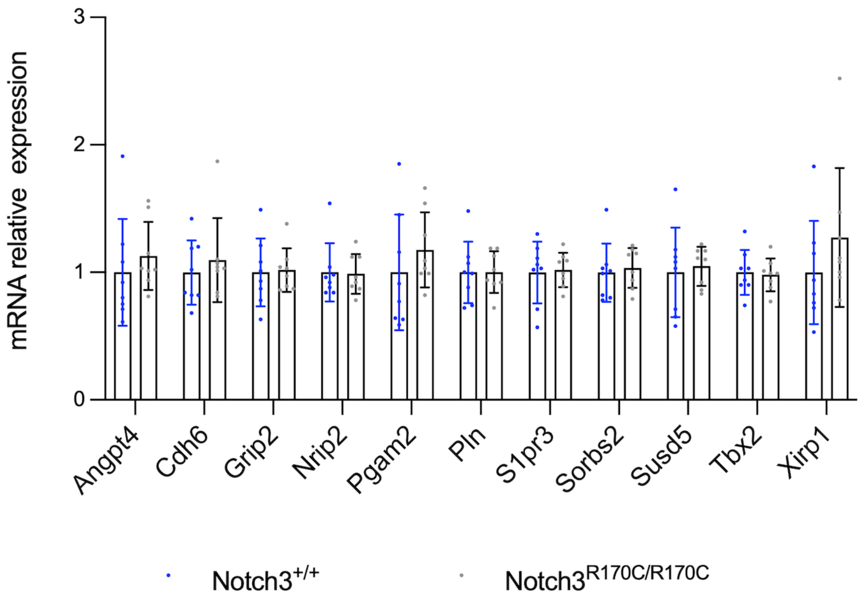

Supplemental Figure 7: **Notch3 activity is unchanged in *Notch3$^{R170C/R170C}$* mice**. Relative mRNA levels for genes regulated by *Notch3* in brain arteries from *Notch3$^{R170C/R170C}$ mice* versus *Notch3$^{+/+}$* mice aged 12 months (n = 8 mice/genotype). Data are reported as a fold-change relative to control mice. Data were analyzed by multiple unpaired t-tests. All adjusted p values are > 0.05.

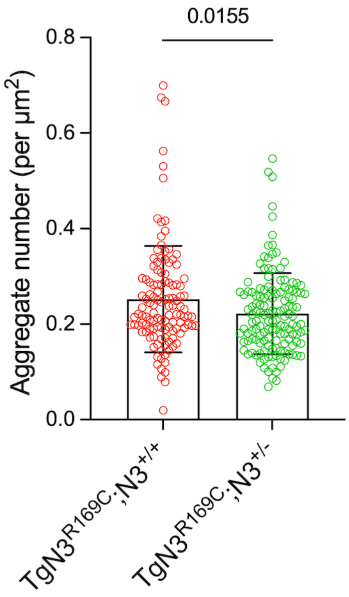

Supplemental Figure 8: Number of Notch3^{ECD} aggregates in arterial SMCs of Tg*Notch3^{R169C}*; *Notch3$^{+/-}$* and Tg*Notch3^{R169C}*; *Notch3$^{+/+}$*. The number of aggregates was quantified in individual arterial SMCs (stringent protocol) of Tg*Notch3^{R169C}*;*Notch3$^{+/+}$* (TgN3^{R169C};N3$^{+/+}$) and Tg*Notch3^{R169C}*;*Notch3$^{+/-}$* (TgN3^{R169C};N3$^{+/-}$) mice aged 6 months (n = 6-7 mice/genotype and 20 SMCs per mouse). Shown are scatter dot plots in which individual points represent individual SMCs. Data were analyzed by unpaired Student's t test with Welch's correction.

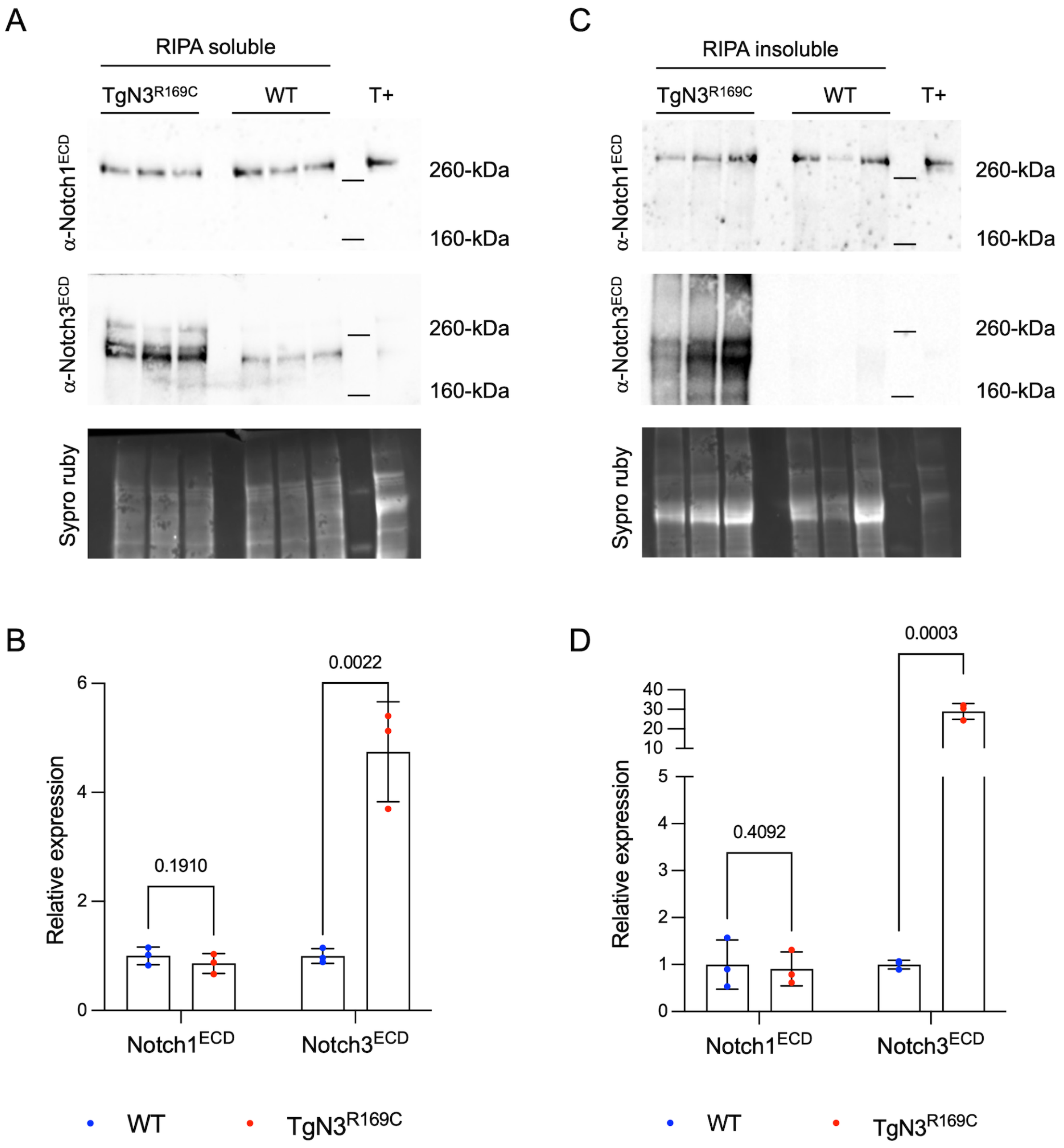

Supplemental Figure 9: Notch1^{ECD}, unlike Notch3^{ECD}, does not accumulate in brain arteries from CADASIL mice. (**A, C**) RIPA soluble (A) and RIPA insoluble (C) fractions were prepared from brain arteries of Tg*Notch3^{R169C}* (TgN3^{R169C}) and wildtype (WT) littermates aged 12 months (n= 3 samples/ genotype, each sample prepared from 2 mice), subjected to SDS-PAGE analysis and transferred to nitrocellulose membranes that were sequentially stained for total proteins (Sypro ruby) (bottom panel), probed with anti-Notch1^{ECD} (top panel) and then Notch3^{ECD} (middle panel) antibodies. A positive control (T+) consisting of protein extracts from 1 month-old wildtype mice was included. (**B, D**) Quantification of Notch1^{ECD} and Notch3^{ECD} protein levels in the RIPA soluble (B) and RIPA insoluble fractions (D) showing that Notch1^{ECD} expression was comparable between wildtype and mutant mice, in both the RIPA soluble and RIPA insoluble fractions, unlike Notch3^{ECD} which accumulated strongly in the RIPA insoluble fraction and to a lesser extent in in the RIPA soluble fraction. Data are reported as a fold-change relative to control mice. Data were analyzed by Student's t-test.

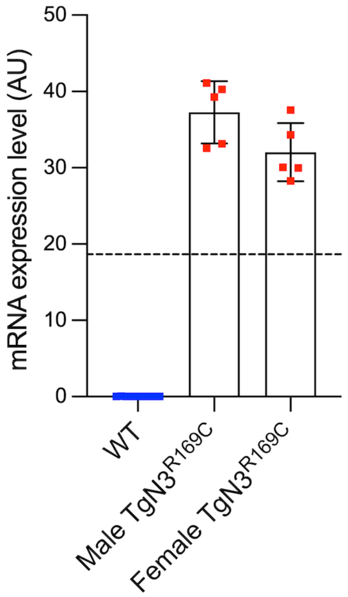

Supplemental Figure 10: The Tg*Notch3*^{R169C} transgene escapes random X inactivation in females. Relative mRNA levels of the transgene in brain arteries dissected from WT and Tg*Notch3*^{R169C} (TgN3^{R169C}) male and female TgN3^{R169C} mice (N = 5 mice/group) were measured by RT-qPCR. Horizontal dotted line represents 50% of the mean transgene expression in transgenic males.
