# Supplementary figures and images for: Protein aggregates containing wild-type and mutant NOTCH3 are major drivers of arterial pathology in CADASIL
Source: J Clin Invest. 2024 Feb 22;134(8):e175789. doi: 10.1172/JCI175789 (PMC11014667; doi:10.1172/JCI175789)

## Slide 1
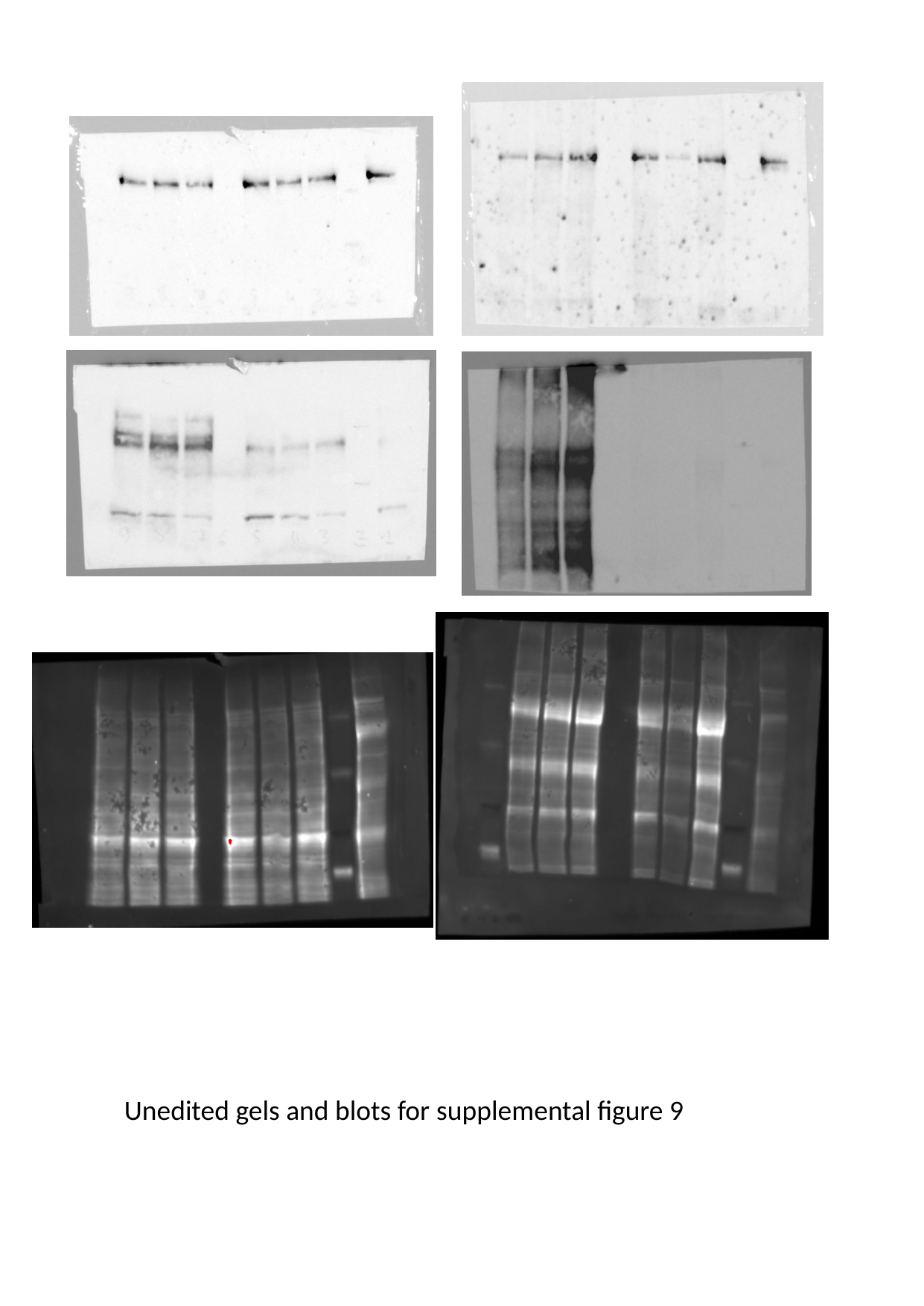

Unedited gels and blots for supplemental figure 9

Supplement: Unedited blot and gel images [file jci-134-175789-s209.pptx]
